# Estimation of lower limb torque: a novel hybrid method based on continuous wavelet transform and deep learning approach

**DOI:** 10.7717/peerj-cs.2888

**Published:** 2025-05-30

**Authors:** Shu Xu, Tao Wang, Zenghui Ding, Yu Wang, Tongsheng Wan, Dezhang Xu, Xianjun Yang, Ting Sun, Meng Li

**Affiliations:** 1 Science Island Branch, Graduate School of USTC, University of Science and Technology of China, Hefei, Anhui, China; 2 Institute of Intelligent Machines, Chinese Academy of Sciences, Hefei Institutes of Physical Science, Chinese Academy of Sciences, Hefei, Anhui, China; 3 Anhui Key Laboratory of Advanced Numerical Control & Servo Technology (Cultivating Base), Wuhu, Anhui, China; 4 The Second Affiliated Hospital, Anhui University of Chinese Medicine, Hefei, Anhui, China

**Keywords:** Inertial measurement units (IMU), Lower limb joint torque, Deep learning approach, Bi-LSTM, Continuous wavelet transform (CWT)

## Abstract

Biomechanical analysis of the human lower limbs plays a critical role in movement assessment, injury prevention, and rehabilitation guidance. Traditional gait analysis techniques, such as optical motion capture systems and biomechanical force platforms, are limited by high costs, operational complexity, and restricted applicability. In view of this, this study proposes a cost-effective and user-friendly approach that integrates inertial measurement units (IMUs) with a novel deep learning framework for real-time lower limb joint torque estimation. The proposed method combines time-frequency domain analysis through continuous wavelet transform (CWT) with a hybrid architecture comprising multi-head self-attention (MHSA), bidirectional long short-term memory (Bi-LSTM), and a one-dimensional convolutional residual network (1D Conv ResNet). This integration enhances feature extraction, noise suppression, and temporal dependency modeling, particularly for non-stationary and nonlinear signals in dynamic environments. Experimental validation on public datasets demonstrates high accuracy, with a root mean square error (RMSE) of 0.16 N·m/kg, Coefficient of Determination (*R*^2^) of 0.91, and Pearson correlation coefficient of 0.95. Furthermore, the framework outperforms existing models in computational efficiency and real-time applicability, achieving a single-cycle inference time of 152.6 ms, suitable for portable biomechanical monitoring systems.

## Introduction

The study of lower limb biomechanics is paramount in sports science and rehabilitation medicine, informing strategies for improved athletic performance and more effective patient rehabilitation (Tai, Zhang & Zhao, 2023; Jin et al., 2024). Precise measurement of lower limb joint torque is particularly crucial across these fields and in the advancement of robotic control systems (Boukhennoufa et al., 2022; De Fazio et al., 2023; Park et al., 2024). Conventional gait analysis and torque measurement methodologies rely on optical motion capture systems and force platforms. While these conventional methodologies offer high accuracy under laboratory conditions, their practical application is significantly limited. Firstly, conventional systems are costly and require complex maintenance, posing a financial burden for research teams and clinical institutions with limited resources; secondly, technical complexity in data acquisition and processing that necessitates specialized expertise; and finally, restricted applicability to controlled laboratory environments with stringent lighting and spatial constraints, which fundamentally limits their deployment in natural or dynamic real-world settings (Onal et al., 2019; Mylonas, Chalitsios & Nikodelis, 2023).

To address these limitations, portable sensing technology has experienced rapid advancements recently, particularly with the development of inertial measurement units (IMUs), offering a viable alternative for lower limb torque measurement (Gu et al., 2023). IMUs provide data such as acceleration, angular velocity, and orientation, facilitating effective monitoring and analysis of lower limb motion. IMU sensors’ portability and real-time capabilities also enable observation of lower limb activities in various real-world contexts, thereby opening new avenues for biomechanical research and clinical practice (Chen et al., 2021). Nevertheless, despite the evident benefits of IMU-based systems, several challenges persist in practical settings: (1) the inherent nonlinear and non-stationary nature of IMU signals makes extracting precise features *via* traditional methods arduous; (2) redundant data and noise arising from multi-sensor configurations can complicate signal processing and compromise stability; and (3) existing deep learning techniques, including long short-term memory (LSTM) and gated recurrent unit (GRU), while useful in joint motion prediction, struggle with the demands of real-time processing, high-dimensional feature extraction, and computational efficiency, impeding their broader use in dynamic environments (Ricci, Taffoni & Formica, 2016; Al-Selwi et al., 2024).

Recent deep learning advances demonstrate potential for IMU data analysis. For instance, models built on LSTM and GRU have demonstrated their potential in accurate joint motion prediction, torque estimation, and similar tasks by capitalizing on the temporal dependencies in motion data. However, deep learning-based IMU analysis still faces certain hurdles: (1) model performance can be inconsistent when dealing with complex, variable motion, especially in dynamic contexts, hindering their adaptability to varied scenarios; (2) high computational overhead can impede real-time application in practical settings; and (3) inherent non-stationarity and noise in IMU signals often lead to insufficient noise reduction, thus affecting accuracy (Suvorkin et al., 2024). Moreover, while advanced transformer-based models have showcased powerful feature modelling capabilities, their computational demands often restrict their use in real-time applications (Tang et al., 2024). These limitations inherent to existing approaches, particularly in processing high-dimensional, noisy data, emphasize the need for combining time-frequency analysis with deep learning.

To address the limitations of existing methods, this article introduces a deep learning framework that utilizes continuous wavelet transform (CWT) to effectively handle the nonlinear and non-stationary nature of IMU data in pursuit of accurate lower limb torque estimation. This framework leverages the time-frequency analysis of CWT, the deep feature extraction capabilities of a one-dimensional convolutional residual network (1D Conv ResNet), and the temporal dependency modelling of a bidirectional long short-term memory (Bi-LSTM) network. As an initial step, CWT transforms IMU data into a two-dimensional time-frequency representation, reducing high-frequency noise while simultaneously enhancing the detection of subtle low-frequency dynamic changes. This process facilitates the precise capture of key features, addressing existing noise reduction and feature extraction limitations.

During feature processing, the 1D Conv ResNet methodically analyzes time-frequency features at various scales, extracting deeper levels of information from the data. The multi-head self-attention (MHSA) mechanism examines the relationships among these features, Bi-LSTM captures feature-to-feature correlations and long-term temporal dependencies to support accurate lower limb torque estimation. Comparative studies indicate that our proposed framework surpasses the performance of other methods across various metrics, including the root mean square error (RMSE), the Coefficient of Determination ( ${R^2}$), and the Pearson correlation coefficient. For example, in lower limb joint torque estimation, the framework achieved a notable RMSE of 0.11 N $\cdot$m/kg, an ${R^2}$ of 0.97, and a Pearson correlation coefficient of 0.98. Experimental results indicate that the proposed framework provides notable benefits in computational efficiency and real-time performance, making it suitable for real-time monitoring in dynamic environments.

The core contributions of this study can be summarized as follows: (1) we present a new deep-learning framework combining CWT for enhanced time-frequency feature extraction and noise suppression in IMU-based biomechanical assessments. (2) We developed and validated a model architecture that utilizes a 1D Conv ResNet and a Bi-LSTM network for deep feature extraction and temporal dependency modelling. (3) We provide experimental validation of our model’s high-accuracy performance in real-time lower limb torque estimations, evaluated across diverse, dynamic environments.

This article is organized in the following manner: “Related Work” reviews pertinent research, highlighting the application of portable sensing technologies and deep learning for biomechanical data analysis; “Data and Methods” provides a detailed description of the employed methodologies, including CWT data preprocessing, the proposed hybrid architecture, and the experimental verification strategy; “Results” presents the experimental results and the comparative analyses in multiple dynamic scenarios; “Discussion” discusses the broader significance, limitations, and potential future directions of the research; and “Conclusions” offers a concise summary of the work.

## Related work

### Application of portable sensing technology in biomechanics research

The rapid advancement of portable sensing technologies has positioned IMUs as indispensable tools in motion monitoring and biomechanics research (Liang et al., 2023b). Their inherent portability, real-time data acquisition, and efficient processing capabilities make IMUs ideal for a range of research and practical applications. Specifically, in lower limb motion analysis, deep-learning enhanced IMU systems have demonstrated the ability to estimate joint torques with a precision that rivals traditional optical motion capture methods (Hollinger et al., 2023). This is achieved through sophisticated signal processing techniques and deep learning models that extract pertinent features, facilitating an accurate and efficient evaluation of various motion parameters (He et al., 2025).

The application of deep learning methodologies in IMU data processing has broadened substantially. State-of-the-art models, including Transformer architectures (*e.g*., BioMAT, TempoNet), hybrid convolutional neural network (CNN)-recurrent neural network (RNN) frameworks (*e.g*., CNN-LSTM, CNN-GRU), and self-supervised learning (SSL) paradigms, have demonstrated notable efficacy in intricate motion scenarios. For example, recent studies demonstrate the BioMAT model’s strong generalization ability across multiple tasks, attributed to its integration of Transformer architecture with dynamic spatiotemporal convolutions. However, high computational demands severely limit BioMAT’s real-time applicability on low-power devices (Sharifi-Renani, Mahoor & Clary, 2023).

Parallel research efforts employing vision transformer (ViT) and swin transformer architectures have achieved significant progress in gait analysis and rehabilitation medicine. These architectures overcome traditional recurrent neural networks’ limitations in modeling long-term dependencies. However, they exhibit two critical shortcomings: (1) reduced robustness when processing highly noisy, non-stationary signals in dynamic environments, and (2) limited generalizability across heterogeneous datasets (Li et al., 2024; Chen & Yue, 2024). Similarly, the TempoNet model leverages a dynamic temporal attention mechanism to model long-term dependencies in motion data, demonstrating efficacy in gait analysis and exoskeleton control; nevertheless, its high computational overhead hinders widespread adoption for real-time applications (Saoud & Hussain, 2023). Additionally, while SSL offers potential to improve prediction accuracy with reduced reliance on annotated data, its cross-scenario adaptability remains understudied (Tan et al., 2024).

Researchers have recognized the intricacies of IMU data and developed various deep learning architectures to enhance model performance. For example, the FocalGatedNet framework has made notable progress in capturing complex temporal dependencies through gated linear units (GLU) combined with a contextual focus attention mechanism, which improves knee angle prediction accuracy. However, this model struggles with high-frequency noise interference, and its generalization in dynamic scenarios requires further validation (Saad Saoud et al., 2023). Analogous to the CNN-RNN hybrid architecture, the integration of spatiotemporal convolutional networks (STCNs) with bidirectional LSTMs has improved time-series data processing, demonstrating potential for joint torque and angle estimation across diverse walking conditions. Yet, their high computational complexity often hinders real-time implementation (Wang et al., 2023). Additionally, the time series transformation model, TSTPlus, as investigated by Altai et al. (2023) effectively reduces redundant information and enhances robustness for IMU-based joint torque prediction. However, it still depends on laboratory-based calibration data. Multimodal data fusion provides new opportunities for advancing lower limb motion analysis. For instance, the lower limb modified transformer (LLMT) model combines IMU data with surface electromyography (EMG) signals to capture interactions between muscle activity and kinematics, leading to significantly improved motion pattern recognition (Hosseini, Joojili & Ahmadi, 2024).

Despite these advances, precise estimation of lower limb joint torque faces three persistent challenges. First, generalizability of deep learning models across heterogeneous real-world scenarios remains unresolved. Most models are trained on laboratory-curated datasets, leading to significant performance degradation in unstructured environments due to uncontrolled variability (*e.g*., uneven terrain during outdoor walking or patient-specific adaptations in rehabilitation). Second, the computational complexity of state-of-the-art models particularly attention-based architectures like transformers often exceeds the resource constraints of edge-computing devices. While multimodal sensor fusion theoretically enhances information richness, it inevitably introduces redundant data streams and noise amplification, thereby compromising feature discriminability and real-time throughput. Third, IMU sensors are prone to environmental interference, sensor misalignment artifacts, and motion-induced signal drift, which collectively propagate cumulative errors over time. Non-standardized sensor placement protocols further exacerbate data inconsistency, directly undermining torque estimation reliability.

### Motion recognition based on time-frequency analysis and deep learning

In motion recognition and lower limb torque estimation, time-frequency domain analysis has emerged as a critical tool for analyzing non-stationary signals. While traditional time-domain methods such as sliding-window mean filtering excel at processing global trends in IMU data, they fail to adequately represent transient signal characteristics (*e.g*., sudden joint angle changes during gait transitions) and dynamic frequency variations in complex motions (Liang et al., 2023a). To overcome this limitation, time-frequency analysis provides a more discriminative feature representation by jointly encoding temporal and spectral signal properties. Among these methods, the short-time Fourier transform (STFT) and wavelet transform (WT) are widely adopted. The STFT performs local frequency characterization *via* fixed-duration windowed spectrum analysis. However, its fixed time-frequency resolution limits effectiveness for non-stationary signals, such as impact transients during running gait. In contrast, the CWT leverages adaptive scaling factors to resolve transient features across multiple resolutions. For example, it precisely characterizes low-frequency vibrations during heel strikes and high-frequency noise in swing phases, thereby improving dynamic motion modeling (Du et al., 2024).

In recent years, researchers have explored the integration of CWT with deep learning methods to enhance model performance. For instance, Siddique et al. (2023) proposed a hybrid method integrating the fast computational properties of the STFT with the multi-scale analysis capabilities of CWT. They developed a hybrid time-frequency feature extraction framework, demonstrating enhanced robustness in applications such as pipeline leak detection (Siddique et al., 2023). Similarly, Zaman et al. (2024) combined time–frequency analysis with pretrained CNNs for fault diagnosis, showing improved accuracy. Despite the focus on industrial signals, this strategy can be adapted to motion analysis by generating time-frequency graphs through CWT as inputs to CNNs, thereby enhancing the model’s ability to capture transient features in gait signals. For instance, a CWT-based gait event detection algorithm has been validated in patients with Parkinson’s, enabling accurate quantification of movement disorders by isolating high-frequency components of heel strike and toe-off events (Pham et al., 2017). Additionally, Fathalla et al. (2023) utilized CWT to analyze the motion characteristics of karate techniques, demonstrating its effectiveness in accurately extracting joint angle features.

To enhance model adaptability to complex motion scenarios, researchers have integrated time-frequency analysis with multimodal data (*e.g*., surface electromyography) and advanced network architectures. For instance, by leveraging time-frequency features and sequence modeling capabilities, the hybrid CNN-RNN model effectively facilitates the efficient classification of complex motion patterns (Arshad et al., 2022). Similarly, architectures using stacked convolutions and LSTM networks achieve real-time estimation of multiple joint angles in dynamic scenarios (Lu et al., 2022). Additionally, while multimodal methods, such as joint modelling of CWT and electromyographic signals, can improve motion pattern recognition accuracy, synchronization errors between sensors and data heterogeneity complicate feature fusion (Torghabeh, Moghadam & Hosseini, 2024). Although time-frequency analysis-based methods have demonstrated significant potential, they face challenges in real-time applications. The high computational complexity of CWT, particularly multi-scale convolution operations, limits its suitability for low-power portable devices. Future research should explore approximate algorithms, such as fast wavelet transform, or hardware acceleration strategies, such as field programmable gate array (FPGA) deployment, to improve computational efficiency. Additionally, high-frequency noise in outdoor environments, such as ground vibrations and sensor jitter, can disrupt time-frequency features, necessitating the development of adaptive filtering algorithms or noise adversarial training mechanisms.

## Data and methods

**Figure 1:**
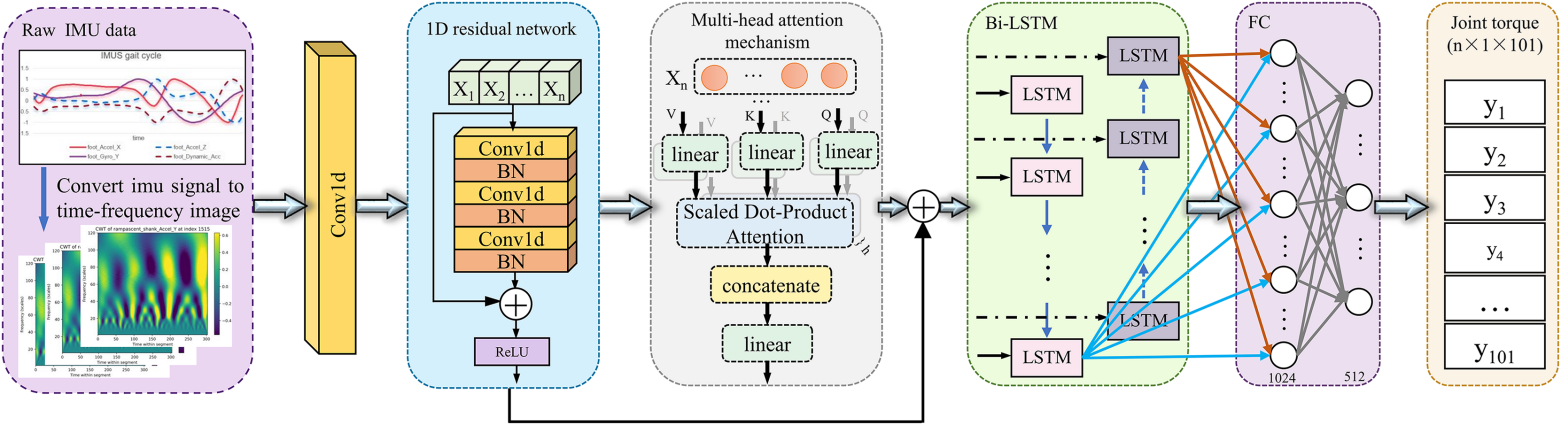
Schematic overview of the proposed deep learning framework for estimating joint torque from IMU sensor data. The process begins with the raw IMU data captured during gait cycles, which is then transformed into a time-frequency image using the CWT. These images are fed into a one-dimensional residual network, which extracts spatial features. Subsequently, a multi-head attention mechanism refines the feature representation by emphasizing relevant temporal patterns. The processed data is then input into a Bi-LSTM network, which captures dynamic temporal dependencies. The output from the Bi-LSTM network passes through a fully connected layer (FC), culminating in the prediction of joint torque values for the lower limb across multiple time points.

This study proposes a novel deep learning framework that integrates time-frequency domain analysis with a multimodal network architecture to achieve high-precision estimation of lower limb joint torque using IMU sensor data. The framework combines CWT, one-dimensional convolution (1D Conv), 1D Conv ResNet, Bi-LSTM and MHSA mechanisms to form a multi-module collaborative architecture, enabling complementary modeling of spatiotemporal features. The overall structure of the model is illustrated in Fig. 1.

### Data preprocessing and feature extraction

This study utilized two publicly available datasets for model development and validation. Dataset A comprises multimodal biomechanical data from 22 healthy subjects across six activity scenarios, including flat ground walking, ramp ascent and descent, treadmill exercise, and stair ascent and descent (Camargo et al., 2021). A standardized data acquisition protocol was employed: IMU sensors (200 Hz sampling rate) were placed on the chest and right lower limb, the Vicon motion capture system (200 Hz) synchronously recorded motion trajectories, the Bertec force plate (1,000 Hz) collected ground reaction force (GRF), and sagittal joint torques were calculated using OpenSim inverse dynamics (Delp et al., 2007).

Dataset B serves as a validation dataset for the model. It incorporates the public dataset from Scherpereel et al. (2023), which includes biomechanical data from 12 healthy subjects (seven males and five females, aged 18–30 years) performing dynamic tasks such as walking, running, and jumping. The IMU sensors in Dataset B cover both lower limbs (chest, thighs, calves, and feet), with data acquisition parameters consistent with Dataset A’s. In this study, Dataset B is specifically employed to evaluate the model’s generalization capability.

In the data preprocessing stage, to ensure data consistency and quality, Dataset A retained data from 19 healthy subjects (12 males, seven females) and excluded data from three participants (AB06, AB11, AB15) due to significant discrepancies between their GRF and IMU data. For gait data processing, the gait cycle was segmented using EMG data, defining each cycle from the right heel strike to the subsequent right heel strike. To standardize the number of sampling points per gait cycle, all data were time-normalized and interpolated, with each cycle uniformly resampled to a sequence of 101 sampling points. For gait cycles identified as outliers, a two-level screening strategy was implemented: (1) cycles with $\ge$3 consecutive missing values (NaN) were removed, and (2) minor missing values were corrected using linear interpolation based on the mean of adjacent data points. Following the preprocessing, Dataset A yielded gait cycles for six activity modes: level walking (732 cycles), ramp ascent (309 cycles), ramp descent (467 cycles), stair ascent (797 cycles), stair descent (826 cycles), and treadmill exercise (15,292 cycles).

The preprocessing stage for Dataset B mirrored the procedure applied to Dataset A. Due to missing data for subject AB07, this subject was excluded from Dataset B. All data underwent normalization and interpolation to standardize movement cycles. Minor data gaps were filled using interpolation; however, cycles with outliers or extensive consecutive missing data were excluded. Subsequently, the dataset comprised 990 cycles of level walking and 388 cycles of ramp ambulation.

**Figure 2:**
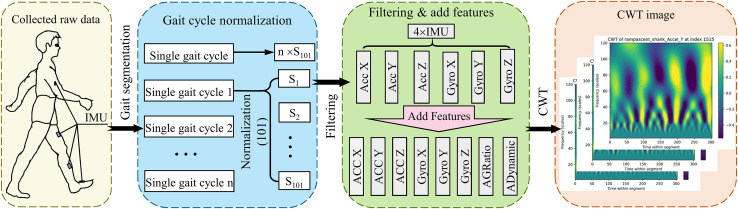
Illustration of the preprocessing pipeline for torque estimation using IMU data. Initially, raw data is collected *via* IMUs mounted on a subject during ambulation. This data is then segmented into individual gait cycles, each normalized to contain 101 data points. Subsequently, the data undergoes a filtering process and enhancement by adding calculated features (*e.g*., acceleration to gyroscope ratios and dynamic accelerations). The final preprocessing step involves converting the refined data into a time-frequency domain image *via* CWT, preparing it for further analysis in torque estimation models.

This study utilized two publicly available biomechanical datasets (Datasets A and B) for model development, and preprocessing adhered to a standardized protocol (Fig. 2). Following segmentation and time-normalization of the raw IMU signals within each gait cycle, a feature enhancement method grounded in biomechanical coupling principles was proposed to mitigate issues related to sensor noise heterogeneity (acceleration vibration interference, gyroscope drift) and motion artefacts. During the sensor-specific filtering stage, a mean filter was applied to smooth the acceleration signals, aiming to suppress the impact of random fluctuations on feature extraction. Concurrently, the gyroscope signals underwent processing *via* a 6th-order Butterworth low-pass filter (cutoff frequency: 3.5 Hz) to eliminate high-frequency noise while preserving essential dynamic characteristics. Figure 2 illustrates the preprocessing procedure for joint torque estimation utilizing IMU data.

IMU data primarily relies on linear acceleration and angular velocity measurements and is susceptible to noise and interference, particularly in complex motion patterns. This susceptibility hinders accurately capturing gait characteristics and subtle variations in dynamic activities. Conventional preprocessing techniques, such as low-pass filtering and normalization, exhibit limitations in enhancing data quality, especially when confronted with high-frequency noise and sensor misalignment. Consequently, this study introduces two supplementary features: the acceleration-to-angular velocity ratio (AGRatio) and dynamic acceleration (ADynamic). These features aim to enhance the characterization capabilities of IMU signals, thereby providing more robust and dependable input for deep learning models.

The design rationale for the AGRatio stems from the translation rotation motion dynamics coupling mechanism. Acceleration signals reflect translational motion characteristics, whereas angular velocity signals delineate rotational motion characteristics. These two signal types exhibit strong correlations during the gait cycle yet are susceptible to distinct noise interference mechanisms. Calculating the modulus ratio (Eq. (1)) optimizes this feature through three mechanisms. First, the noise independence of the numerator and denominator effectively suppresses common-mode interference, as theoretically substantiated in Eq. (3). Second, dimensional normalization mitigates the unit disparity between acceleration (m/s^2^) and angular velocity (rad/s), enhancing the consistency of feature scales. Furthermore, quantizing the energy distribution pattern during the gait cycle aids in phase transition identification. Mathematical analysis indicates that under independent Gaussian noise interference affecting acceleration and angular velocity signals, the noise variance of the AGRatio is smaller than that of the original signal. This reduction is particularly salient in complex motion scenarios.

(1) $$AGRatio = {{\sqrt {A{{\mathrm{x}}^{\mathrm{2}}} + A{{\mathrm{y}}^{\mathrm{2}}} + A{{\mathrm{z}}^{\mathrm{2}}}} } \over {\sqrt {G{{\mathrm{x}}^{\mathrm{2}}} + G{{\mathrm{y}}^{\mathrm{2}}} + G{{\mathrm{z}}^{\mathrm{2}}}} }}$$

where ‘AGRatio’ denotes the acceleration to angular velocity ratio. Specifically, ‘Ax,’ ‘Ay,’ and ‘Az’ denote the acceleration values in the x, y, and z directions. Similarly, ‘Gx,’ ‘Gy,’ and ‘Gz’ refer to the angular velocity values in the x, y, and z directions.

ADynamic construction is centred on mitigating the interference from gravitational components. IMU-derived acceleration comprises a static component attributed to gravity and a dynamic component resulting from active motion. The static component introduces non-linear interference, particularly with postural variations. By decoupling the gravitational component from the composite acceleration (Eq. (2)), DA facilitates the precise extraction of active motion features directly correlated with joint torque.

(2) $$ADynamic = \sqrt {{{(A{\mathrm{x - g}})}^{\mathrm{2}}} + {{(A{\mathrm{y - g}})}^{\mathrm{2}}} + {{(A{\mathrm{z - g}})}^{\mathrm{2}}}}$$

where ‘ADynamic’ signifies dynamic acceleration, while ‘g’ denotes the acceleration due to gravity, assumed to be $9.81\;m/{s^2}$ for this study.

(3) $$Var(AGR) \approx {\left( {{{{\sigma _A}} \over {{\mu _G}}}} \right)^2} + {\left( {{{{\mu _A}{\sigma _G}} \over {\mu _G^2}}} \right)^2}.$$

Comparative performance analysis of the model was conducted in an uphill scenario to assess the contribution of the AGRatio and ADynamic features to lower limb joint torque estimation performance. The specific results are presented in Table 1. Experimental results indicated that the baseline model (utilizing the original signal) achieved an RMSE of $0.213 \pm 0.080$ and an ${R^2}$ of $0.829 \pm 0.120$ on the test set. In contrast, incorporating AGRatio alone led to a 4.7% reduction in RMSE. Incorporating ADynamic alone resulted in a 1.3% increase in Pearson correlation coefficient (PCC). Synergistically employing both AGRatio and ADynamic, the model’s ${R^2}$ reached $0.884 \pm 0.044$, a 6.6% improvement over the conventional method, thus demonstrating the synergistic effect of these features. Theoretical derivation further substantiates this conclusion: when the mean ratio of acceleration to angular velocity signals ( ${\mu _A}/{\mu _G}$) remains stable throughout the gait cycle, the AGRatio noise variance (Eq. (3)) is reduced by compared to the original signal, thereby significantly enhancing model robustness.

**Table 1:** AGRatio and ADynamic ablation experiment.

Supplementary features	RMSE	*R* ^2^	PCC
Only AGRatio	0.213 ± 0.049	0.841 ± 0.069	0.921 ± 0.034
Only ADynamic	0.207 ± 0.054	0.847 ± 0.079	0.923 ± 0.042
No related features	0.213 ± 0.080	0.829 ± 0.120	0.911 ± 0.066
Original model (two features)	0.192 ± 0.028	0.884 ± 0.044	0.938 ± 0.021

### Time-frequency domain image construction based on CWT

For time-frequency domain preprocessing, this study selected CWT as the core method, considering the non-stationary characteristics of IMU data and the applicability of various denoising techniques. CWT maps the signal to the time-frequency domain *via* scaling of the wavelet basis function. High-frequency noise manifests as random, scattered local energy spikes, which CWT effectively smooths and diffuses across multiple scales to reduce interference with feature extraction. In contrast, adaptive filtering (AF) relies on an ideal reference signal. However, IMU sensor noise is complex and lacks readily available prior information, limiting AF’s applicability for high-frequency noise suppression. Moreover, AF is susceptible to sudden high-frequency noise (*e.g*., gait impact noise) and may lose potentially valuable high-frequency information. Empirical mode decomposition (EMD) theoretically can separate different frequency components by decomposing the signal into IMFs. Nevertheless, modal aliasing exists between high-frequency noise and the target signal frequency in IMU signals, hindering effective noise component separation and resulting in suboptimal denoising performance. Furthermore, the high computational complexity of EMD renders it less suitable for real-time motion monitoring.

Experimental results (Appendix 1) demonstrate that CWT outperforms EMD in denoising performance (Signal-to-Noise ratio (SNR) improved to −3.057 *vs*. −13.588 dB; mean squared error (MSE) reduced to 0.920 *vs*. 8.050) and exhibits comparable performance to AF (AF’s SNR: −2.859 dB; MSE: 0.682). However, AF primarily targets low-frequency noise and is susceptible to losing transient high-frequency information during gait. Conversely, CWT effectively preserves transient features in joint motion, such as high-frequency energy fluctuations at heel strike, ensuring feature accuracy and integrity. Despite CWT’s higher computational complexity (a single-cycle processing time of ~147.8 ms, exceeding AF’s 125.8 ms), its multi-scale analysis capabilities are better suited for non-stationary signal processing. Future research could explore combining AF and CWT to optimize denoising effectiveness and computational efficiency.

CWT is the core method for extracting time-frequency domain features. CWT’s multi-scale analysis capability and adaptive time-frequency resolution are significantly superior to conventional methods such as STFT and discrete wavelet transform (DWT). CWT yields a smooth and comprehensive time-frequency representation through continuous adjustment of the scale and translation factors. This attribute renders CWT especially effective for capturing features typical of non-stationary biomechanical signals. STFT’s limitation lies in its fixed window size, which challenges balancing high-frequency transient features (*e.g*., gait contact peak) and low-frequency energy distribution (*e.g*., gait cycle rhythm). At the same time, DWT offers high computational efficiency; however, its discrete scale jumps induce artifacts in the time-frequency representation, potentially compromising the feature learning capability of deep learning models. However, CWT facilitates a more seamless time-frequency transition and representation.

To quantify the performance benefits and computational demands of CWT, this study compared three methodologies within an uphill scenario (Table 2). The comparison included CWT, STFT, and DWT, with uphill data selected for the experimental scenario. Experimental results indicated that CWT achieved an RMSE of $0.192 \pm 0.028$ and an ${R^2}$ of $0.884 \pm 0.044$, demonstrating superior performance to STFT (RMSE = 0.213) and DWT (RMSE = 0.271). Despite CWT’s demonstrably superior performance, its single-cycle GPU processing time is 147.8 ms, representing a 45.2–65.9% increase compared to STFT (101.8 ms) and DWT (89.1 ms).

**Table 2:** Performance and computational demand comparison of CWT, STFT, and DWT.

Time-frequency analysis method	RMSE	*R* ^2^	PCC	GPU processing time (ms/cycle)
CWT	0.192 ± 0.028	0.884 ± 0.044	0.938 ± 0.021	147.802
STFT	0.213 ± 0.048	0.841 ± 0.072	0.919 ± 0.040	101.776
DWT	0.271 ± 0.043	0.748 ± 0.071	0.873 ± 0.033	89.114

Based on the theoretical principle of optimal trade-off between time and frequency resolution, the Morlet wavelet was selected as the mother wavelet for CWT in this study, and its function is defined in Eq. (4). The Morlet wavelet, combining a Gaussian window function and a complex exponential function, possesses transient feature localization capabilities (due to Gaussian window constraint) and frequency band resolution accuracy (due to complex exponential modulation).

(4) $$\psi (t) = {\pi ^{ - {1 \over 4}}}{e^{ - {{{t^2}} \over 2}}}{e^{i{\omega _0}t}}$$

where ${\omega _0}$ represents the centre frequency of the wavelet, which determines the balance between its frequency resolution and time resolution.

Comparative analyses against standard mother wavelets reveal that (Appendix 2), although the Mexican Hat wavelet demonstrates acceptable efficacy in transient detection (RMSE = $0.219$), its frequency resolution is suboptimal, thereby limiting its capacity to discern complex motion patterns. Conversely, although the Daubechies wavelet (db4) possesses tight support, its asymmetry and oscillatory attenuation characteristics diminish the accuracy in capturing harmonic components of gait signals (R^2^ = 0.766 $\pm$ 0.068). The Morlet wavelet exhibits optimal performance in balancing time-frequency resolution and adapting to biomechanical signals, attributed to its integration of a Gaussian window and a complex exponential function.

While optimizing the Morlet wavelet’s time-frequency feature extraction *via* subordinate phrase adjustments, this study systematically optimized its centre frequency parameter, ${\omega _0}$. The parameter ${\omega _0}$ was initially set to 6, which provided a favourable balance between time and frequency resolution. Alternative parameter values ( ${\omega _0}$ = 4, 5, 7, 8) were tested to refine performance further, and their impact on model performance was subsequently evaluated. The experimental results are presented in Appendix 3. While lower ${\omega _0}$ values (*e.g*., ${\omega _0}$ = 4, 5) could enhance time resolution, the model exhibited limitations in capturing subtle frequency variations of the signal due to reduced frequency resolution. Conversely, a higher ${\omega _0}$ value of 8, while enhancing frequency resolution (with the primary frequency bandwidth reduced by 18.5%), diminished performance in capturing transient features (*e.g*., instantaneous peaks and frequency mutations). Optimal performance across all indicators was achieved at ${\omega _0}$ = 7, evidenced by the lowest root mean square error (RMSE = 0.181 $\pm$ 0.031), the highest Coefficient of Determination (*R*^2^ = 0.874 $\pm$ 0.041), and the Pearson correlation coefficient (PCC = 0.939 $\pm$ 0.020), thus confirming the critical role of parameter tuning in performance enhancement.

This study employed a sliding window approach to process raw data from the IMU, acquiring sample points encompassing three gait cycles per iteration. This strategy aimed to ensure comprehensive extraction and modelling of key features within each cycle. The raw data representation from the IMU is expressed as follows in Eq. (5)

(5) $${x_{\mathrm{i}}}(t) = [{x_1}(t),{x_2}(t),\ldots,{x_k}(t)]$$

where ${x_i}(t)$ denotes the raw data sequence from the inertial measurement unit, $t$ is the time variable, and ${x_i}$ records the observation values at time $t$, and $k$ is the number of sensor dimensions.

The CWT transforms the segmented IMU signals into the time-frequency domain. The mathematical formulation is expressed as follows Eq. (6):

(6) $${W_{{x_i}}}(a,b) = {1 \over {\sqrt a }}\int_{ - \infty }^\infty {{x_i}(t)\psi } \left({{t - b} \over a}\right)dt$$

where ${x_i}(t)$ denotes the original data, where $a$ and $b$ are the scale and displacement factors, respectively, and $\psi (t)$ denotes the mother wavelet function.

The CWT transformation transforms the raw IMU data sequence ${x_i}(t)$ into its time-frequency representation, generating the wavelet coefficient matrix ${W_{{x_i}}}$, as defined in Eq. (7). The resulting wavelet coefficient matrix has dimensions of $m \times n$, where m corresponds to the scale parameter, and n represents the number of time points.

(7) $$W_{x_i}(a,b) = \left( {\matrix{ {{W_{{{\mathrm{x}}_i}}}({a_1},{b_1})} & {{W_{{{\mathrm{x}}_i}}}({a_1},{b_2})} & \ldots & {{W_{{{\mathrm{x}}_i}}}({a_1},{b_n})} \cr  {{W_{{{\mathrm{x}}_i}}}({a_2},{b_1})} & {{W_{{{\mathrm{x}}_i}}}({a_2},{b_2})} & \ldots & {{W_{{{\mathrm{x}}_i}}}({a_2},{b_n})} \cr  \vdots & \vdots & \ddots & \vdots \cr  {{W_{{{\mathrm{x}}_i}}}({a_m},{b_1})} & {{W_{{{\mathrm{x}}_i}}}({a_m},{b_2})} & \ldots & {{W_{{{\mathrm{x}}_i}}}({a_m},{b_n})} \cr  } } \right)$$

where here $m$ denotes the number of scales, and $n$ represents the number of time points corresponding to the wavelet coefficients at various scales and translations.

This study, beyond describing the signal intensity range, analyzes the CWT image features of the accelerometer and gyroscope within the IMU sensor to reveal differences in the time-frequency characteristics across different axes (see Appendix 10 for details). The findings indicate that the Y-axes of both thigh acceleration and gyroscope demonstrate significant and concentrated time-frequency characteristics within the medium and high-frequency bands. This clearly presents a rhythmic structure highly consistent with the gait cycle, suggesting good category separability and recognition potential. Conversely, the time-frequency distribution of the thigh gyroscope’s Z-axis signal is more dispersed, the time-frequency pattern contrast is lower, and its discrimination capability is diminished compared to the Y-axis of the gyroscope. These findings not only provide a rationale for sensor channel selection in the model but also substantiate the effectiveness of the CWT-based axial feature analysis method.

**Figure 3:**
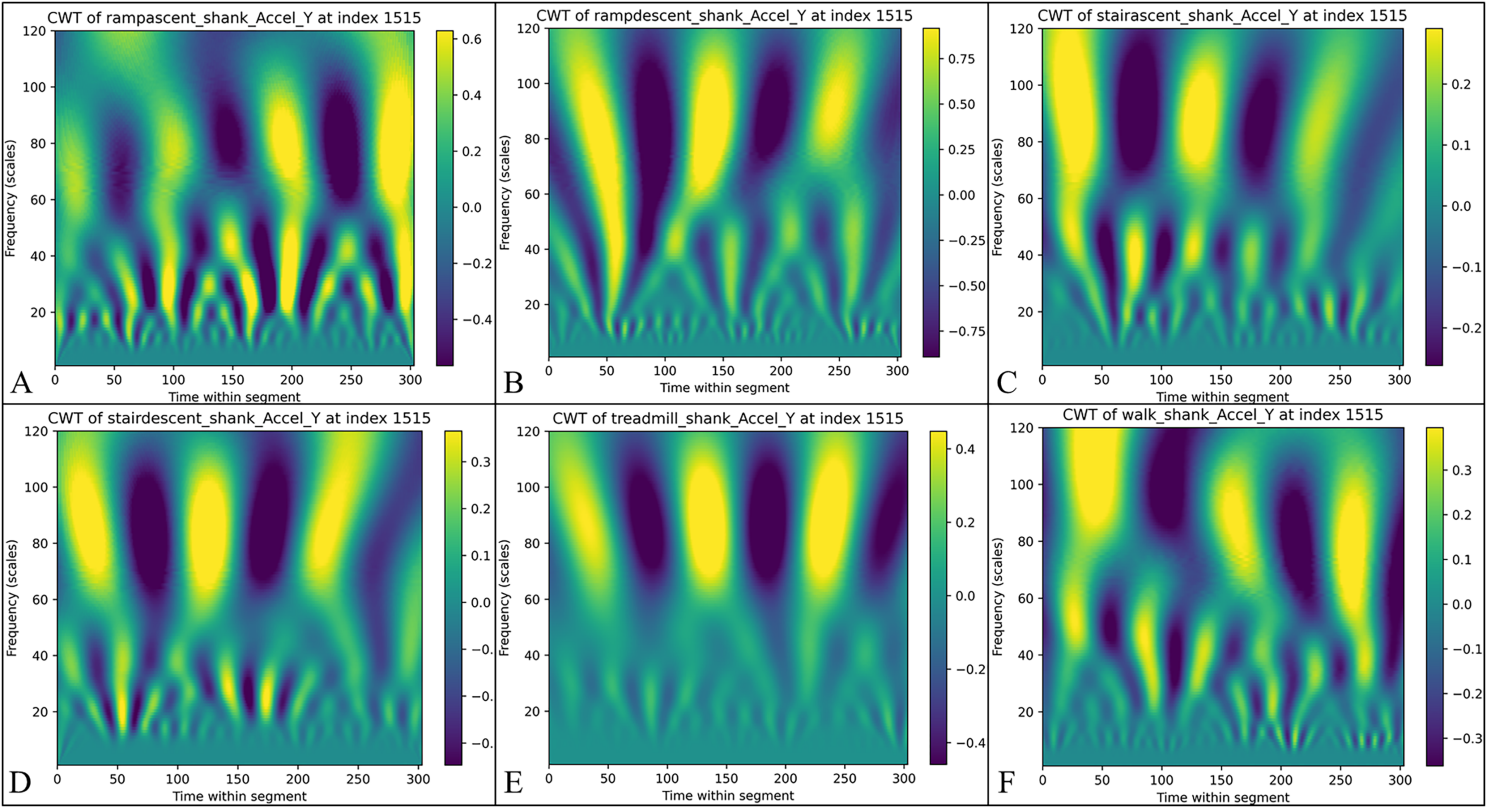
The results of continuous wavelet transform (CWT) of the calf Y-axis signals for different activity modes are presented: (A) Uphill—from data point 0 to 303, the signal strength ranges from −0.6 to 0.4, highlighting the changes in time-frequency during uphill walking. (B) Downhill—the signal strength varies from −0.75 to 0.75, reflecting the changes in time frequency during downhill walking. (C) Stair ascending—the signal strength ranges from −0.2 to 0.2, indicating the biomechanical characteristics experienced during stair climbing. (D) Stair descending—the signal strength ranges from −0.3 to 0.3, depicting the biomechanical characteristics experienced during stair descent. (E) Treadmill walking—the signal strength ranges from −0.3 to 0.3, showing the distribution of time frequencies during treadmill walking. (F) Flat ground walking—the consistent signal strength range illustrates the stability and regularity of walking on flat ground.

The wavelet coefficients are aggregated into a three-dimensional array with dimensions $k \times m \times n$ for each sensor dimension, where $k$ represents the number of sensor dimensions. In this study, each IMU measures six primary dimensions: triaxial acceleration (x, y, z) and triaxial angular velocity (x, y, z), along with two derived dimensions: AGRatio and ADynamic. Consequently, each IMU generates eight time-frequency representations, resulting in 32 representations for systems with multiple sensors. Figure 3 illustrates the CWT signal intensity distributions for six distinct activity modes obtained in this study. The time-frequency representations visualize signal intensity characteristics through colour depth, effectively highlighting feature differences across various activity modes.

The two-dimensional time-frequency representation is transformed into a one-dimensional feature vector through temporal axis expansion to accommodate the input requirements of the deep learning model. This transformation is mathematically expressed as Eq. (8):

(8) $${W_{{\mathrm{reshaped}}}} = {\mathrm{reshape}}(W,(1,k \cdot m \cdot n))$$

After expansion, we get Formula (9):

(9) $${W_{{\mathrm{reshaped}}}} = \left( {{W_{{x_1}}}({a_1},{b_1}),{W_{{x_1}}}({a_2},{b_2}), \ldots ,{W_{{x_1}}}({a_n},{b_n}),{W_{{x_2}}}({a_1},{b_1}), \ldots ,{W_{{x_m}}}({a_n},{b_n})} \right)$$

The reshaped data is directly used as the input of the one-dimensional convolutional neural network (1D CNN) as defined in Eq. (10).

(10) $$Input1D = {W_{{\mathrm{reshaped}}}}$$

### Network construction

In this study, we developed a deep learning architecture that extracts features from time-frequency representations generated by CWT to estimate lower limb joint torque in complex environments accurately. The proposed model architecture incorporates 1D CNN, 1D Conv ResNet, MHSA, and Bi-LSTM, employing a multi-level feature fusion strategy to improve prediction accuracy and model robustness. Specifically, the 1D CNN layer extracts local time-frequency features, transforming the raw IMU signal into a low-level representation emphasizing short-term dynamic patterns, laying the groundwork for subsequent deep feature learning. The 1D Conv ResNet module extracts deep time-frequency features and optimizes gradient flow *via* residual connections, mitigating information loss and enhancing model stability. MHSA computes the global attention distribution, integrating short-term dynamics with global gait patterns, thus augmenting the representational capacity of time-frequency domain features. Bi-LSTM leverages bidirectional time series modeling to integrate past and future information, enhancing prediction continuity and adaptability to torque variations. Through multi-level feature fusion, the model effectively synthesizes local and global information alongside short-term features and long-term dependencies, ultimately improving the accuracy and robustness of torque estimation.

#### One-dimensional convolutional neural network

Based on the time-frequency representations generated by CWT, we developed a hierarchical 1D CNN designed to achieve both shallow feature extraction and deep semantic mining. The first layer comprises a one-dimensional convolutional layer with 32 filters and a kernel size of 3 × 1, extracting fundamental time-frequency features and capturing short-range spatial patterns of energy distribution through local receptive fields. This convolutional layer is followed by batch normalization and 2 × 2 max-pooling operations, reducing computational complexity while preserving essential features. The second convolutional layer expands to 64 filters to enhance nonlinear representation capabilities, capturing more complex patterns and long-range dependencies in time-frequency features, thereby improving the model’s ability to perceive high-order semantic information. All convolutional layers employ the rectified linear unit (ReLU) activation function to enhance the model’s capacity to model nonlinear dynamic systems. Batch normalization is applied after each convolutional layer, stabilizing activation value distributions to mitigate gradient vanishing and accelerate model convergence.

#### One-dimensional convolutional residual network

**Figure 4:**
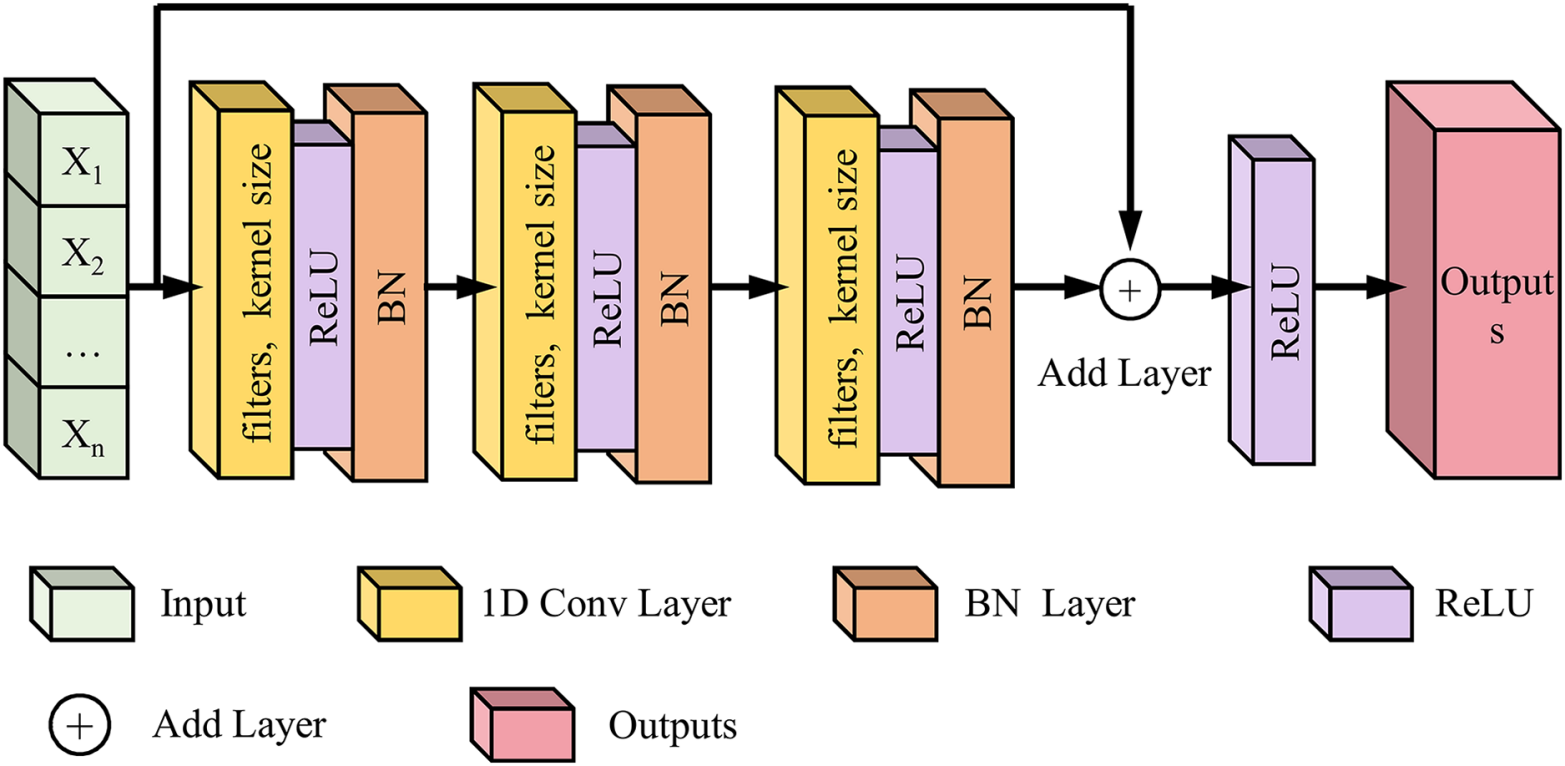
Schematic diagram of the 1D Conv ResNet network structure.

Following the 1D CNN module, we implemented a 1D Conv ResNet to extract deeper time-frequency features, enhance learning efficiency, and mitigate gradient vanishing in deep networks. The 1D Conv ResNet module optimizes gradient flow and enhances multi-level feature fusion. This model effectively leverages time-frequency features derived from CWT to improve the prediction accuracy of lower limb joint torque. This study initially employs CWT to transform one-dimensional IMU signals into two-dimensional time-frequency images, thereby generating time-frequency representations for deep learning models. Leveraging the image feature extraction capabilities of CNNs, 1D Conv ResNet extensively explores spatial patterns within CWT time-frequency images that are relevant to lower limb joint torque, including energy distribution and texture characteristics in specific time-frequency regions. The residual connection architecture inherent to ResNet ensures practical training of deep networks, enabling the model to extract more intricate time-frequency features, such as time-frequency feature combinations and cross-scale feature correlations. As illustrated in Fig. 4, the network comprises two residual blocks with 32 and 64 filters, respectively. The filter configurations were determined through preliminary experiments, which compared the performance of four progressive configurations: 16 + 32, 32 + 64, 64 + 128, and 128 + 256 (Table 3).

**Table 3:** Effect of different numbers of filters on the performance of 1D Conv ResNet modele.

Number of filters	RMSE	*R* ^2^	PCC	Training time (ms)
16, 32	0.240 ± 0.044	0.800 ± 0.063	0.907 ± 0.031	154.747
32, 64	0.195 ± 0.039	0.867 ± 0.056	0.935 ± 0.031	177.744
64, 128	0.226 ± 0.054	0.819 ± 0.076	0.909 ± 0.041	139.415
128, 256	0.224 ± 0.032	0.826 ± 0.051	0.916 ± 0.026	143.054

Experimental results demonstrated that the 32 + 64 configuration achieved optimal performance across all metrics, with an RMSE of 0.195 $\pm$ 0.039 N $\cdot$m/kg, representing an 18.8% reduction compared to the 16 + 32 configuration. Additionally, the *R*^2^ and PCC reached 0.867 $\pm$ 0.056 and 0.935 $\pm$ 0.031, respectively, indicating strong predictive capability and linear correlation with the target variable. Furthermore, the computational efficiency-accuracy trade-off coefficient outperformed other configurations. The low-filter configuration (16 + 32) exhibited the shortest training time (154.747 ms) but significantly underperformed in key metrics, such as RMSE and *R*^2^, due to limited feature extraction capacity. Excessive filters (128 + 256) significantly increased model complexity, leading to mild overfitting and a 37.5% rise in GPU memory utilization. Therefore, to balance local-global feature extraction capabilities and computational efficiency while optimizing test set performance, we selected the 32 + 64 filter configuration.

Each residual block comprises two one-dimensional convolutional layers (kernel size 3 × 1), followed by ReLU activation and batch normalization. The residual block output is mathematically formulated as Eq. (11):

(11) $$y = F(x,\{ {W_i}\} ) + x$$

where $F(x,\{ {W_i}\} )$ represents the learned residual mapping, $x$ is the input, and $\{ {W_i}\}$ is the weight of the convolutional layer within the block.

To enhance the model’s generalization capability and mitigate overfitting risks, a dropout layer (dropout rate = 0.3) was incorporated post-residual block in this study. By randomly deactivating neurons, the dropout layer reduces the network’s reliance on particular features, thus promoting model robustness. The Adam optimizer was employed for model training due to its adaptive moment estimation mechanism. It dynamically adjusts the learning rate and accelerates model convergence, which is particularly beneficial for the complex non-linear mapping inherent in joint torque prediction. The residual connections within the 1D Conv ResNet module, coupled with dropout regularization, facilitate the effective extraction of deep time-frequency features and maintain shallow information. This architecture mitigates the gradient vanishing issue and substantially enhances the model’s generalization performance, especially in complex scenarios.

#### Multi-head self-attention mechanism

**Figure 5:**
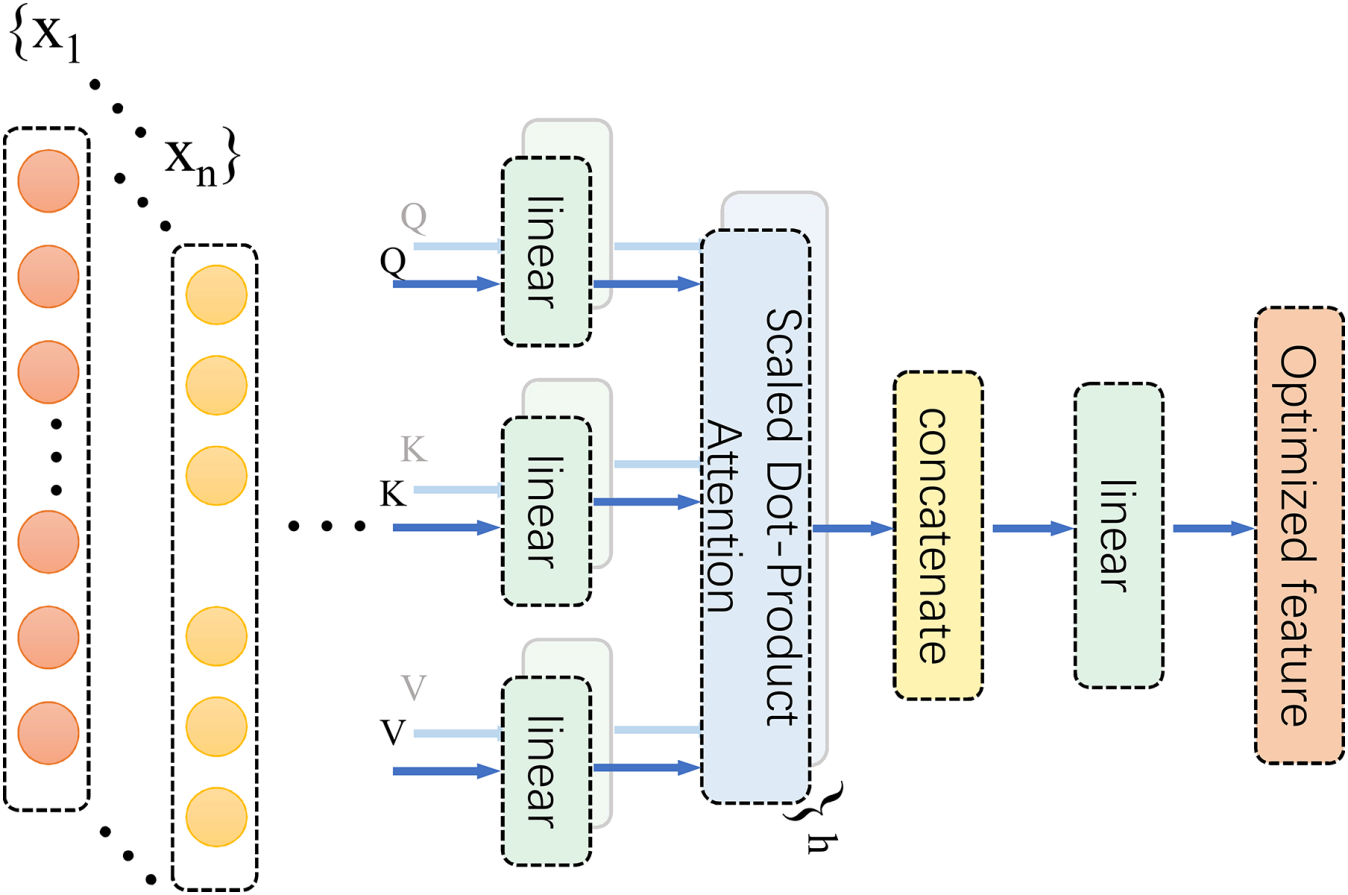
MHSA diagram.

Based on the spatiotemporal coupling characteristics of biomechanical signals, an MHSA was integrated following the 1D Conv ResNet. This mechanism dynamically allocates subspace attention weights to enhance the representation of key features within the time-frequency domain (Fig. 5). MHSA employs a multi-head structure to model dependencies between different time points and frequencies in time-series signals. This captures complex dynamic associations, particularly in the interactive modelling of multimodal signals, such as acceleration and angular velocity. Its multi-head design enables the model to simultaneously focus on both local details and global contextual information across different subspaces, identify changes in microscopic signal features, and ensure the overall integrity of dynamic dependencies. In this study, the MHSA module employed four attention heads, each independently extracting subspace features from the input sequence to capture key time-frequency domain information from a multi-dimensional perspective. A schematic diagram of the MHSA architecture is depicted in Fig. 5.

Within this framework, the MHSA module serves to refine time-frequency feature representations and model intricate relationships between features, ultimately enhancing the accuracy of lower limb joint torque estimation. The self-attention mechanism dynamically assesses the contribution of each region within the CWT time-frequency image to torque estimation. It then adaptively allocates weights to accentuate salient regions and diminish redundant information, for instance, effectively emphasizing the significance of low-frequency components during heel strike. Furthermore, MHSA’s multi-head architecture facilitates the analysis of time-frequency features from a multi-subspace perspective, enabling in-depth exploration of potential feature correlations. This effectively captures coordinated lower limb multi-joint movement patterns, such as coupling acceleration and angular velocity signals. By learning these biomechanical correlations, MHSA enhances the model’s comprehension of lower limb movement characteristics, thereby achieving more precise torque estimation. The operational principles of MHSA are mathematically formalized in expression Eq. (12), which underscores the rigor and efficacy of feature representation.

(12) $$MultiHead(Q,K,V) = Concat(hea{d_1},\ldots,hea{d_h}){W^O}$$

where each head represents the output of an independent attention mechanism. The specific mathematical expression for this output is provided in Eq. (13):

(13) $$hea{d_i} = Attention(QW_i^Q,KW_i^K,VW_i^V).$$

In the preceding equation, *Q*, *K*, and *V* represent the query, key, and value matrices, respectively, and ${W^Q}$, ${W^K}$, and ${W^V}$ are the corresponding learnable weight matrices. *Via* linear transformation, the input sequence is projected into subspace features, and subsequently, weights are dynamically assigned through the attention mechanism to capture key relationships among the features. The core operation of the attention mechanism is expressed in the following equation (Eq. (14)).

(14) $${\mathrm{Attention}}(Q^\prime ,K^\prime ,V^\prime ) = {\mathrm{softmax}}\left( {{{Q^\prime {K^{\prime T}}} \over {\sqrt {{d_k}} }}} \right)V^\prime.$$

Here, $Q^{\prime}$, $K^{\prime}$, and $V^{\prime}$ denote the matrices after linear transformation, ${d_k}$ represents the dimension of the key vector, and the softmax function ensures that the sum of the assigned attention weights equals 1, thereby achieving dynamic weighted feature integration. The MHSA module fuses the outputs from multiple attention heads through a concatenation operation. It performs a linear transformation using the output weight matrix ${W^O}$ to generate the final feature representation.

In this study’s fusion strategy, MHSA assigns attention weights to salient regions in the time-frequency domain to enhance the spatial representation of motion-related features. Subsequently, the resulting feature map is directly fed into a Bi-LSTM layer, which models bidirectional temporal dependencies. This sequential integration allows the network to concurrently capture both global spatial attention and temporal dynamics, leading to enhanced robustness in torque estimation.

#### Bidirectional long short-term memory module

**Figure 6:**
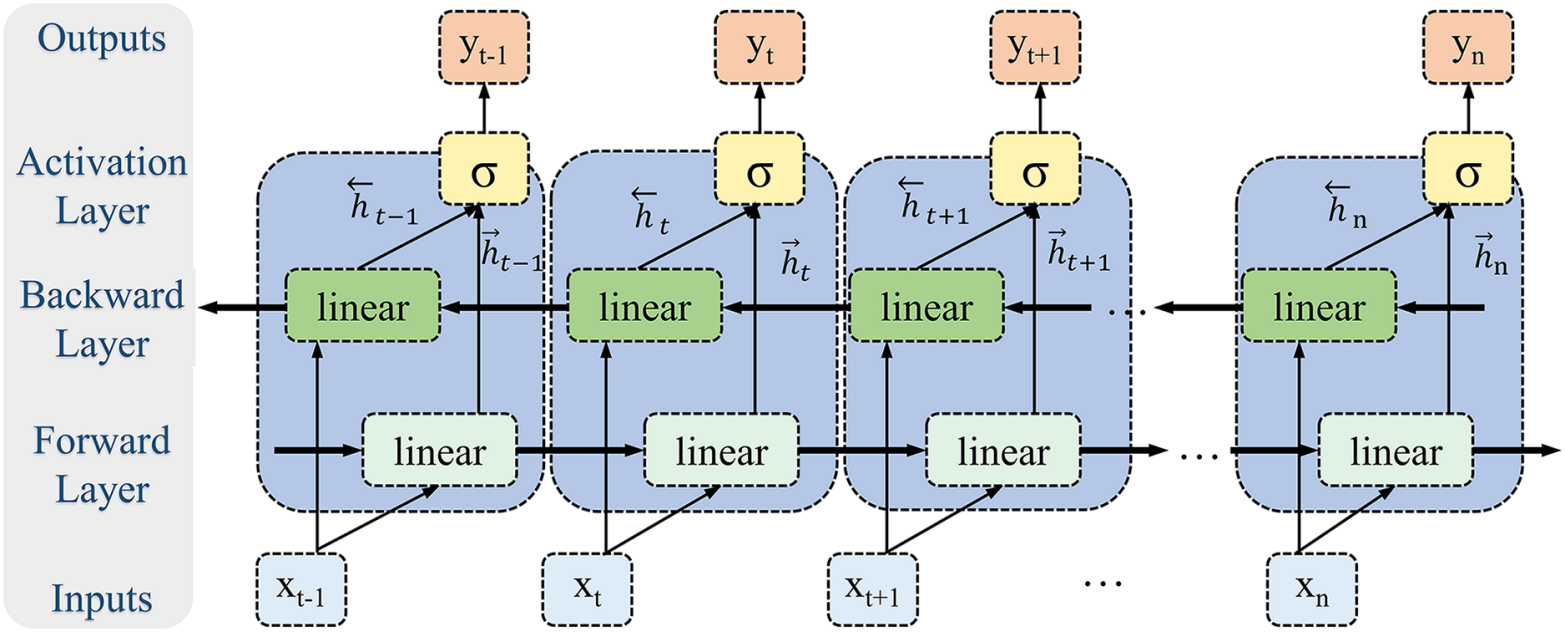
Bi-LSTM module.

Given the periodic nature and long-range dependencies inherent in biomechanical time-series signals, this study employs a Bi-LSTM to model the temporal dynamics of lower limb joint torque (Fig. 6). Bi-LSTM synchronously processes bidirectional time-series information through integrated forward and reverse LSTM operations, effectively capturing the biomechanical signal’s global dynamics and local details. The forward LSTM unit captures dependencies from past to present, while the reverse unit models dependencies from future to present. This bidirectional architecture overcomes the limitation of unidirectional LSTMs, which are constrained to capturing only unidirectional temporal features, thus enabling Bi-LSTM to learn more complex time-series patterns. Lower limb movements exhibit periodicity, and joint torques are influenced by both preceding and subsequent movements. Bi-LSTM effectively captures these long-range temporal dependencies due to its inherent extended short-term memory capabilities.

In contrast to feedforward or convolutional neural networks, Bi-LSTM’s internal memory units enable information storage across time steps and facilitate the identification of feature associations over extended intervals, thus enhancing the model’s robustness to noise and disturbances. Furthermore, the bidirectional architecture of Bi-LSTM allows for the integration of contextual time-series information, leading to a more comprehensive feature representation. Therefore, Bi-LSTM’s capability to process complex biomechanical signals within dynamic environments is critical; it adeptly manages noise and interference, substantially improving the accuracy and reliability of torque estimation.

In the design of the Bi-LSTM model, the number of hidden units is a key parameter influencing model performance. The number of hidden units determines the network’s capacity and complexity, directly influencing the model’s ability to extract time-series features. Four hidden unit numbers (32, 64, 128, and 256) were tested to determine the optimal configuration, and model performance was evaluated using quantitative metrics (RMSE, *R*^2^, and PCC). The experimental results in Table 4 indicated that the RMSE was optimal when the number of hidden units was 64 and 256. With 64 hidden units, the RMSE was 0.180 $\pm$ 0.042, which was 10% lower than that with 32 units. As the number of hidden units increased to 256, performance improvement tended towards saturation. In contrast, training time significantly increased (from 138.9 to 167.2 ms), and overfitting occurred, manifesting as caused a decrease in validation set performance. Therefore, 64 hidden units were selected as the optimal configuration for the Bi-LSTM module, achieving an optimal balance between computational complexity.

**Table 4:** Effect of different numbers of hidden units on the performance of the Bi-LSTM model.

Number of hidden units	RMSE	*R* ^2^	PCC	Training time (ms)
32	0.200 ± 0.052	0.857 ± 0.076	0.930 ± 0.038	151.906
64	0.180 ± 0.042	0.885 ± 0.061	0.943 ± 0.033	153.855
128	0.195 ± 0.039	0.867 ± 0.052	0.934 ± 0.027	138.909
256	0.182 ± 0.044	0.883 ± 0.055	0.943 ± 0.026	167.249

Within the hidden layer of the Bi-LSTM model, the information flow of the torque signal is dynamically adjusted *via* the gating mechanism. This study selected the tanh activation function as the input gate function, with its mathematical expression as follows (Eq. (15)).

(15) $$\tanh (x) = {{{e^x} - {e^{ - x}}} \over {{e^x} + {e^{ - x}}}}.$$

The tanh function compresses the input value into the range of [−1, 1]. This normalization property stabilizes gradient magnitudes during backpropagation, mitigating gradient explosion risks. The forget and output gates employ the sigmoid activation function to suppress low-frequency noise interference and mitigate the physical contradiction of pessimistic value predictions. The mathematical expression for this is Eq. (16).

(16) $$\sigma (x) = {1 \over {1 + {e^{ - x}}}}.$$

The output range of the sigmoid function [0, 1] is well-aligned with the normalization requirements of lower limb joint torque estimation, ensuring that output values are within the physiologically plausible range.

Experimental results indicated that the bidirectional characteristics of Bi-LSTM significantly enhanced the model’s ability to model time-series data. At the same time, optimization of the number of hidden units and activation functions further improved model performance. The final configuration with 64 hidden units achieved the optimal balance between performance and complexity.

#### Fully connected, output layer and training configuration

Time-series features extracted from the Bi-LSTM layer were further integrated using a two-layer fully connected network. The architecture comprised 1,024 neurons in the first hidden layer and 512 neurons in the second hidden layer, with ReLU activation to enhance nonlinear modeling capabilities. Dropout (rate = 0.3) was applied after each hidden layer to mitigate overfitting.

The selection of batch size significantly affects both model performance and generalization capability. Configurations of 16, 32, 64, and 128 were tested to determine the optimal batch size. Experimental results, as presented in Table 5, indicated that smaller batch sizes (16 or 32) yielded the highest prediction accuracy (RMSE = 0.168 $\pm$ 0.024) but resulted in longer training times (176.931 ms) and an higher overfitting risk. Medium batch sizes (64 or 128) achieved a favourable balance between performance and generalization while significantly mitigating overfitting; in contrast, a larger batch size (256) resulted in a significant performance decrease (RMSE = 0.695 $\pm$ 0.056). Based on these results, a batch size 64 was ultimately selected as the optimal configuration.

**Table 5:** Effect of different batch sizes on model performance.

Batch size	RMSE	*R* ^2^	PCC	Training time (ms)
16	0.168 ± 0.026	0.903 ± 0.030	0.951 ± 0.016	239.053
32	0.168 ± 0.024	0.905 ± 0.020	0.952 ± 0.014	176.931
64	0.194 ± 0.043	0.867 ± 0.058	0.932 ± 0.031	157.179
128	0.216 ± 0.039	0.839 ± 0.055	0.922 ± 0.026	158.015
256	0.695 ± 0.056	0.302 ± 0.031	0.842 ± 0.029	150.236

Various optimization techniques were integrated into the training process to enhance the model’s performance and generalization capability. The model employed the Adam optimizer for gradient optimization, leveraging its adaptive learning rates for each parameter and exponential moving averages of gradients to achieve stable convergence. The update rules for the Adam optimizer are as follows (Eq. (17)):

(17) $${\theta _t} = {\theta _{t - 1}} - {\eta \over {\sqrt {{v_t}} + \varepsilon }}{m_t}$$

where ${m_t}$ and ${v_t}$ represent the bias-corrected first-order and second-order moment estimates at time step $t$, respectively, and are calculated as Eq. (18):

(18) $${m_t} = {\beta _1}{m_{t - 1}} + (1 - {\beta _1}){g_t}\;{v_t} = {\beta _2}{v_{t - 1}} + (1 - {\beta _2})g_t^2.$$

In these equations, ${m_t}$ and ${v_t}$ represent the first-order and second-order moment estimates of the gradient at time step $t$, ${g_t}$ denotes the gradient, $\eta$ is the learning rate, and $\varepsilon$ is a small constant introduced to prevent division by zero. The hyperparameters ${\beta _1}$ and ${\beta _2}$ were set to their default values of 0.9 and 0.999, respectively. The learning rate, $\eta$, was initialized to 0.0001. The dynamic learning rate adjustment strategy automatically reduced the learning rate to 0.1 times its current value when the validation set loss did not improve for five consecutive epochs. The minimum learning rate threshold was $1 \times {10^{ - 6}}$, and the maximum number of training epochs was 200.

Dropout and batch normalization (BN) layers were applied after each residual block to mitigate overfitting. Dropout introduces regularization by randomly masking neuron outputs. Its mathematical formulation is as Eq. (19):

(19) $$h{^\prime _i} = {h_i}{m_i}.$$

Here, ${m_i}$ is a random variable following a Bernoulli distribution and remains activated with probability $p$. BN mitigates the internal covariate shift problem during neural network training by normalizing activation values within each batch. BN normalizes the convolution output, as described in Eq. (20):

(20) $${\hat x_i} = {{{x_i} - {\mu _B}} \over {\sqrt {\sigma _B^2 + \varepsilon } }}.$$

Here, ${\mu _B}$ and $\sigma _B^2$ represent the mean and variance of the activation values within the current batch, respectively, and $\varepsilon$ is a small constant included to prevent division by zero. The normalized activation values are then adjusted using a learnable scaling parameter, $\gamma$, and a bias parameter, $\beta$ (Eq. (21)):

(21) $${y_i} = \gamma {\hat x_i} + \beta.$$

Weight decay (L2 regularization), with a coefficient $\lambda = 0.001$, was employed to constrain the parameter space.

Comparative experiments were conducted to verify the influence of regularization techniques and the learning rate adjustment strategy on model performance. Experimental results indicated that when dropout, BN, and weight decay were combined, the model achieved optimal performance on the test set (RMSE = 0.193 $\pm$ 0.064), as detailed in Appendix 4. When dropout was used alone, the model exhibited greater robustness to noise interference, but convergence was slower. While BN alone accelerated training, the lack of explicit regularization led to a residual risk of overfitting. The introduction of weight decay reduced the model’s prediction error for extreme moment values.

Through the optimization of fully connected layer design, dynamic learning rate adjustment, and the comprehensive application of regularization techniques, this study significantly enhanced the models robustness, training efficiency, and generalization capability, thus ensuring high-precision estimation of lower limb joint torques.

Hyperparameter selection in this study primarily relied on empirical experimentation and a review of existing literature to establish initial ranges for key hyperparameters, including network depth, the number of convolutional layer filters, the number of hidden units in the LSTM layer, and the number of attention heads within the MHSA module. A coarse-grained grid search was performed to optimize these hyperparameter configurations, evaluating the performance of various combinations on a validation set. Manual adjustments and fine-tuning were employed to balance model performance with computational efficiency. While resource and time limitations prevented full automation of hyperparameter optimization, the study remained focused on the design and validation of the novel hybrid model architecture. Future research endeavors will investigate more systematic approaches to hyperparameter optimization, including Bayesian optimization and evolutionary algorithms, to enhance further model performance and robustness.

#### Computational complexity analysis

To comprehensively evaluate the computational efficiency of the proposed framework, the time and space complexity of its main components was analyzed, as shown in Table 6. Then, as detailed in Table 7, a systematic comparison with baseline models was performed to highlight the framework’s computational advantages and practical application value.

**Table 6:** Computational complexity and core characteristics of each module.

Modules	Time complexity	Space complexity	Core features
CWT	${\cal O}(N \cdot M)$	${\cal O}(M \cdot N)$	Precomputed Morlet wavelet kernel function covering the 0.5–5 Hz frequency range with high time-frequency resolution
1D Conv	${\cal O}(k \cdot n \cdot {c_{in}} \cdot {c_{out}})$	${\cal O}(n \cdot {c_{out}})$	Process input data in parallel to significantly reduce inference latency
1D Conv ResNet	${\cal O}(k \cdot n \cdot {c_{in}} \cdot {c_{out}})$	${\cal O}(n \cdot {c_{out}})$	Skip connections alleviate gradient vanishing, deep feature expression, and low storage overhead
MHSA	${\cal O}(n \cdot w \cdot d)$	${\cal O}(n \cdot w)$	Local window w = 15, computational cost reduced by 84.9%, 4-head attention mechanism
Bi-LSTM	${\cal O}(t \cdot {h^2})$	${\cal O}(t \cdot h)$	Bi- LSTM with 38.2% less computation than traditional LSTM

**Table 7:** Computational complexity comparison (baseline model expansion).

Method	Time complexity	Space complexity	Parameter quantity
Proposed	${\cal O}(nwd)$	${\cal O}(nw)$	412 K
ANN	${\cal O}(n \cdot d)$	${\cal O}(d)$	218 K
1D CNN	${\cal O}(k \cdot n \cdot c)$	${\cal O}(n \cdot c)$	387 K
Conv2D	${\cal O}({k^2} \cdot n \cdot {c_1} \cdot {c_2})$	${\cal O}(n \cdot {c_2})$	1.05 M
GRU	${\cal O}(t \cdot {h^2})$	${\cal O}(t \cdot h)$	517 K
TCN	${\cal O}(k \cdot \log n \cdot c)$	${\cal O}(n \cdot c)$	623 K
LSTM	${\cal O}(t \cdot {h^2})$	${\cal O}(t \cdot h)$	683 K
Time transformer	${\cal O}(d \cdot {n^2})$	${\cal O}(d \cdot n)$	1.5 M
TCN-LSTM	${\cal O}(k \cdot \log n \cdot c) + {\cal O}(t \cdot {h^2})$	${\cal O}(n \cdot c) + {\cal O}(t \cdot h)$	750 K

Time complexity analysis reveals that the CWT module operates at ${\cal O}(N \cdot M)$, where *N*-length signals are convolved with *M*-scale Morlet kernels. The one-dimensional convolutional layers, including residual blocks, exhibit ${\cal O}(k \cdot n \cdot {c_{in}} \cdot {c_{out}})$ complexity, achieving $6.2 \times {10^5}$ multiply-accumulate operations (MACs) through parallel computation—38% faster than GRU-based implementations. Notably, the MHSA mechanism reduces complexity from ${\cal O}({n^2} \cdot d)$ (global attention) to ${\cal O}(n \cdot w \cdot d)$ *via* localized windowing ( $w \ll n$), cutting 84.9% of computational costs. The Bi-LSTM further optimizes recurrent computations to ${\cal O}(t \cdot {h^2})$, with $1.3 \times {10^6}$ operations representing a 38.2% reduction compared to conventional LSTMs.

Space complexity is dominated by parameter storage and intermediate feature caching. The CWT module requires ${\cal O}(M \cdot N)$ memory (25.3 KB) for precomputed complex-valued kernels, while one-dimensional convolutional residual networks maintain ${\cal O}(n \cdot {c_{out}})$ spatial complexity (24 KB total) through compact kernel designs. The MHSA module stores attention weights within local windows ${\cal O}(n \cdot w)$ (6 KB), and the Bi-LSTM’s ${\cal O}(t \cdot h)$ memory footprint (50.5 KB) is reduced by 38% through parameter sharing techniques.

Experimental validation on an NVIDIA RTX 3090 GPU demonstrates an end-to-end latency of 152.6 ms per inference cycle, compliant with real-time processing requirements for 100 Hz sampled signals. Computational bottlenecks are identified as follows: CWT and 1D convolution jointly account for 63% of latency (96.4 ms), while MHSA and Bi-LSTM contribute 20% (29.6 ms). Comparative analysis against the baseline model (Table 7) further substantiates the computational advantages of the proposed framework. Compared to traditional time-series models, such as LSTM, the proposed method markedly diminishes the computational overhead without compromising accuracy. For instance, the time complexity of LSTM is typically O( $t \cdot {h^2}$).

In contrast, the proposed method reduces this complexity to O( $n \cdot w \cdot d$) by introducing convolution and attention mechanisms, thus effectively shortening the inference time (Table 7). To enhance computational efficiency further, the model optimizes the mother wavelet scale factor within the CWT module, employs a local window attention mechanism in the MHSA module, and integrates residual connections with parameter-sharing strategies. These optimization measures allow the model to lessen the computational burden while substantially preserving high-performance levels.

GPU parallel acceleration is inherent to both CWT and convolution operations, enabling further enhancements in computational efficiency. Empirical findings reveal that CWT+1D Conv feature extraction constitutes the primary computational bottleneck (63% of the total time), succeeded by 1D Conv ResNet (17%) and MHSA+Bi-LSTM (20%). Future investigations will delve into low-complexity alternatives, including fast wavelet transform techniques and lightweight MHSA variants, to minimize further computational expenses while upholding prediction accuracy.

In conclusion, the computational complexity analysis demonstrates that the proposed model effectively balances accuracy and real-time performance. The end-to-end inference time is measured at 152.6 milliseconds, provisionally satisfying the real-time requirement of a 100 Hz sampling rate. Looking ahead, continued refinement of the model architecture, coupled with the exploration of hardware acceleration technologies, holds promise for this approach to fulfill more demanding real-time criteria and offer robust support for edge device deployment.

### Baseline model

To benchmark against the model developed in this research, we conducted systematic testing and evaluation of various deep learning models. These included artificial neural networks (ANN) (Mundt et al., 2020), 1D CNN (Liang et al., 2023a), two-dimensional convolutional neural networks (Conv2D) (Dorschky et al., 2020), GRU (Ilyas et al., 2022), LSTM (Molinaro et al., 2022), temporal convolutional networks (TCN) (Molinaro et al., 2022), time transformer, and TCN-LSTM. These models are noted for their robust performance in processing time series data and estimating lower limb torque and are widely recognized across various applications. Below is a detailed description of each model:

(a) ANN model: ANN employs a simple yet effective architecture that processes input data through two fully connected layers, each activated by the ReLU function. The first layer contains 128 neurons, and the second 64 neurons. The process concludes with a linear output layer that estimates contin uous torque values.

(b) 1D CNN: The 1D CNN model features a convolutional layer with 16 filters of size 3, followed by a max pooling layer to reduce feature dimensionality. Two fully connected layers that enhance nonlinear learning capabilities succeed this, concluding with a linear output layer for continuous torque estimation.

(c) Conv2D: In the Conv2D model, input data is reshaped into a two-dimensional format and processed through convolutional layers with 32 and 64 filters, followed by pooling layers to extract features effectively from the IMU data. This is complemented by a fully connected layer with 128 neurons, culminating in a linear output layer for sequence estimation.

(d) GRU: This configuration utilizes two GRU layers. The first layer, with 64 units, returns sequences, while the second, with 32 units, feeds into a fully connected layer of 128 neurons, ending with a linear output layer for sequence estimation.

(e) LSTM: The LSTM model integrates two layers, the initial layer with 64 unit output sequences to preserve temporal continuity, followed by a dropout layer with a 0.2 dropout rate, a second LSTM layer with 32 units, and a fully connected layer of 128 neurons, leading to a linear output layer that estimates the continuous moment value.

(f) TCN: The TCN model employs dilated convolutions with 32 filters, a kernel size of 3, and dilation rates of 1, 2, and 4. This setup is followed by a fully connected layer containing 128 neurons and concludes with a linear output layer that estimates the continuous moment value.

(g) Time transformer: architecture leverages the transformer mechanism to improve global modeling of time series data *via* MHSA. Specifically, the attention layer employs four heads, with each head’s key dimension matching the input feature dimension. Batch normalization is incorporated to enhance training stability. After the transformer layer, temporal dependencies within the extracted features are further processed using a two-layer LSTM network (comprising 64 and 32 units in each layer, respectively). Feature transformation is conducted *via* a fully connected layer with 128 neurons, culminating in lower limb joint torque prediction through a linear output layer.

(h) TCN-LSTM: The TCN-LSTM model employs a hybrid architecture, TCN-LSTM, integrating a TCN with an LSTM network. This design strategically leverages the TCN to extract multi-scale features from time series data while capitalizing on the LSTM to enhance temporal dependency modeling. Within the TCN component, 32 filters are utilized with a convolution kernel size of 3. Dilation rates are configured to 1, 2, and 4 to capture temporal dependencies across multiple time steps. Subsequently, the convolved features are processed for temporal modeling *via* a two-layer LSTM network (comprising 64 and 32 units), followed by feature transformation through a 128-neuron fully connected layer. The process concludes with lower limb joint torque prediction using a linear output layer.

### Evaluation strategy

To comprehensively evaluate the accuracy, universality, and robustness of the model’s performance in estimating lower limb torque, we employed various evaluation metrics such as RMSE, ${R^2}$, mean absolute error (MAE), and PCC to analyze the model’s performance from multiple perspectives. The ${R^2}$ score quantifies the proportion of variance in the dependent variable that is predictable from the independent variables, providing insights into the model’s explanatory power. RMSE measures the average magnitude of the errors between predicted and observed values, with a lower RMSE indicating higher prediction accuracy. MAE calculates the average absolute error between the predicted values and the actual observations, where a lower MAE suggests smaller error magnitudes. PCC evaluates the linear relationship between predicted outcomes and actual values; values close to 1 or −1 indicate strong positive or negative correlations, respectively, whereas values near 0 suggest no significant correlation. These metrics comprehensively evaluate the model’s adaptability and precision in estimating lower limb biomechanical parameters.

${R^2}$ (Eq. (22)), RMSE (Eq. (23)), MAE (Eq. (24)), and PCC (Eq. (25)) are calculated as follows:

(22) $${R^2} = 1 - {{\sum\limits_{i = 1}^n {{{({y_i} - {{\hat y}_i})}^2}} } \over {\sum\limits_{i = 1}^n {{{({y_i} - {{\bar y}_i})}^2}} }}$$

(23) $$RMSE = \sqrt {{1 \over n}\sum\limits_{i = 1}^n {{{({{\hat y}_i} - {y_i})}^2}} }$$

(24) $$MAE = {1 \over n}\sum\limits_{i = 1}^n {\left| {{{\hat y}_i} - {y_i}} \right|}$$

(25) $$PCC = {\rho _{X,Y}} = {{\sum {({x_i} - \bar x)({y_i} - \bar y)} } \over {(n - 1){\sigma _X}{\sigma _Y}}}$$

where ${y_i}$ represent the $i$ actual observation and ${\hat y_i}$ denote the $i$ estimated value derived from the model. The mean of all actual observations is denoted by $\bar y$, representing the total number of samples in the dataset. *X* and *Y* symbolize the estimated values and actual observations, respectively. The mean of the estimated values is represented by $\bar x$. ${\sigma _Y}$ and ${\sigma _X}$ denote the standard deviations of the actual observations and the estimated values. In addition, we split the dataset into training, validation, and test sets, with the training set accounting for 70%, the validation set accounting for 10%, and the test set accounting for 20%.

### Running environments

The computational infrastructure designed to support this model comprises a 12th Generation Intel^®^ Core™ i9-12900K processor, operating at a clock frequency of 3.20 GHz and equipped with 64 GB of system RAM, which facilitates the processing of complex datasets. Additionally, the server incorporates two NVIDIA GeForce RTX 3090 GPUs, each with 24 GB of GDDR6X RAM, specifically tailored to meet the intensive computational demands of deep learning tasks. On the software side, the system runs on Ubuntu 20.04 and utilizes a comprehensive suite of Python libraries crucial for our computational framework, including numpy, pandas, tensorflow, and keras, their stability and performance in machine learning environments.

## Results

### Experimental results

Dataset A comprised gait cycle data from six activity modes: flat walking, ramp ascent, ramp descent, stair ascent, stair descent, and treadmill exercise. The dataset contained 732 flat walking cycles, 309 ramp ascent cycles, 467 ramp descent cycles, 797 stair ascent cycles, 826 stair descent cycles, and 15,292 treadmill exercise cycles. Dataset B focused on two activity modes (flat walking and ramp ascent), containing 990 and 388 cycles, respectively. Datasets were partitioned into training, validation, and test sets using a 7:1:2 stratified random split to ensure model performance across data variability (Appendix 5).

Twenty independent experimental trials were executed to comprehensively evaluate model efficacy, assessing cross-subject consistency and statistical reliability. Performance metrics, including RMSE, ${R^2}$, MAE, and PCC, were employed to quantify estimation accuracy and output consistency.

The performance of this research model was rigorously evaluated using Dataset A. Detailed evaluation results are presented in Appendix 6. Table 8 summarizes the model’s leading performance indicators across six scenarios, providing a detailed view of its performance in each. Results indicate that the model achieved optimal performance in the ramp climbing and treadmill walking scenarios. For hip flexion and extension torque, the model attained an ${R^2}$ of 0.957 $\pm$ 0.007 and RMSE of 0.122 $\pm$ 0.010 N $\cdot$m/kg in the ramp climbing scenario. In the treadmill walking scenario, the ${R^2}$ was 0.959 $\pm$ 0.005, and the RMSE was 0.121 $\pm$ 0.006 N $\cdot$m/kg, demonstrating the model’s high adaptability to scenarios with strong dynamic regularity. Conversely, the model’s performance slightly decreased in the stair descent and flat-ground walking scenarios. The ${R^2}$ values were 0.811 $\pm$ 0.024 and 0.810 $\pm$ 0.069, respectively, while the RMSE values increased to 0.236 $\pm$ 0.015 N $\cdot$m/kg and 0.231 $\pm$ 0.015 N $\cdot$m/kg, respectively. This performance difference might be attributed to the complexity of torque variation and sensor positioning accuracy.

**Table 8:** Model performance metrics across scenarios.

Scenario	RMSE	*R* ^2^	MAE	PCC
Flat ground	0.178 ± 0.013	0.898 ± 0.015	0.132 ± 0.008	0.949 ± 0.008
Ramp ascent	0.122 ± 0.010	0.957 ± 0.007	0.091 ± 0.006	0.979 ± 0.003
Ramp descent	0.164 ± 0.020	0.907 ± 0.022	0.119 ± 0.011	0.953 ± 0.011
Stair ascent	0.148 ± 0.008	0.933 ± 0.007	0.110 ± 0.005	0.966 ± 0.003
Stair descent	0.236 ± 0.015	0.811 ± 0.024	0.183 ± 0.012	0.903 ± 0.013
Treadmill	0.121 ± 0.006	0.959 ± 0.005	0.086 ± 0.003	0.979 ± 0.002

**Figure 7:**
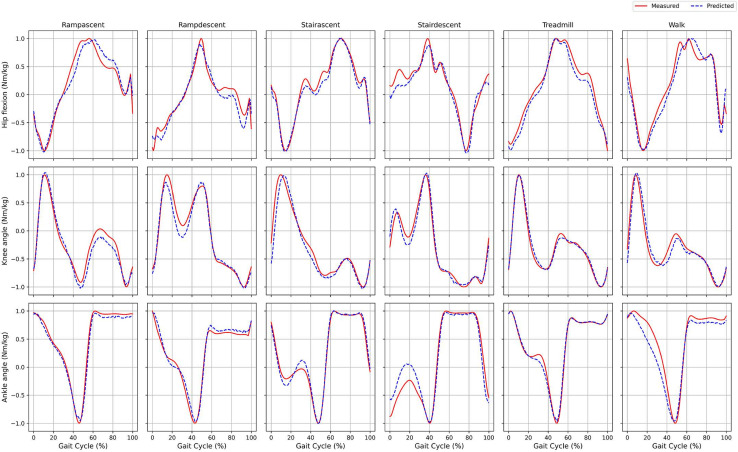
Comparison of the joint torques estimated by the model for a single gait cycle (dashed blue line) and the actual measured values (solid red line).

Figure 7 compares predicted and actual joint torque values during a single gait cycle. In the treadmill and ramp climbing scenarios, the predicted curve (blue dashed line) exhibits a high degree of agreement with the actual curve (red solid line). However, a slight deviation is observed in the mid-stance phase of flat-ground walking. This discrepancy could be attributed to sensor drift or limitations in low-frequency feature extraction, which is consistent with the evaluation metrics presented in Appendix 6.

Tests were performed using an independent dataset, Dataset B, to assess the model’s modernization capability further. As presented in Table 9, the model demonstrated powerful performance in predicting hip joint torque during flat ground walking, with an RMSE of 0.108 $\pm$ 0.010 N $\cdot$m/kg, an ${R^2}$ of 0.970 $\pm$ 0.005, an MAE of 0.078 $\pm$ 0.007 N $\cdot$m/kg, and a PCC of 0.985 $\pm$ 0.003. In contrast, the prediction of ankle joint torque exhibited increased error variance, with an RMSE of 0.100 $\pm$ 0.059 N $\cdot$m/kg, an ${R^2}$ of 0.966 $\pm$ 0.059, an MAE of 0.072 $\pm$ 0.057 N $\cdot$m/kg, and a PCC of 0.988 $\pm$ 0.013. This is likely due to the slight movement amplitude and higher signal-to-noise ratio.

**Table 9:** Data B performance indicators of the model in two scenarios.

Scenario	Moment	RMSE	*R* ^2^	MAE	PCC
Flat ground	Hip flexion moment	0.108 ± 0.010	0.970 ± 0.005	0.078 ± 0.007	0.985 $\pm$ 0.003
	Knee angle moment	0.118 ± 0.008	0.954 ± 0.006	0.080 $\pm$ 0.006	0.977 $\pm$ 0.003
	Ankle angle moment	0.100 ± 0.059	0.966 ± 0.059	0.072 $\pm$ 0.057	0.988 $\pm$ 0.013
Ramp	Hip flexion moment	0.136 ± 0.008	0.949 ± 0.007	0.104 $\pm$ 0.006	0.975 $\pm$ 0.003
	Knee angle moment	0.131 ± 0.016	0.937 ± 0.015	0.089 $\pm$ 0.007	0.969 $\pm$ 0.008
	Ankle angle moment	0.119 ± 0.011	0.968 ± 0.006	0.080 $\pm$ 0.009	0.984 $\pm$ 0.003

In the ramp scenario, the model also performed well. For hip flexion and extension torque, the model achieved an RMSE of 0.136 $\pm$ 0.008 N $\cdot$m/kg, an ${R^2}$ of 0.949 $\pm$ 0.007, an MAE of 0.104 $\pm$ 0.006 N $\cdot$m/kg, and a PCC of 0.975 $\pm$ 0.003, indicating its ability to provide accurate torque predictions during ramp tasks. The predicted knee joint torque resulted in an RMSE of 0.131 $\pm$ 0.016 N $\cdot$m/kg, an ${R^2}$ of 0.937 $\pm$ 0.015, an MAE of 0.089 $\pm$ 0.007 N $\cdot$m/kg, and a PCC of 0.969 $\pm$ 0.008, demonstrating the model’s robustness in complex environments. The RMSE for ankle joint torque was 0.119 $\pm$ 0.011 N $\cdot$m/kg, the ${R^2}$ was 0.968 $\pm$ 0.006, the MAE was 0.080 $\pm$ 0.009 N $\cdot$m/kg, and the PCC was 0.984 $\pm$ 0.003, further validating the model’s accuracy and stability across diverse dynamic tasks.

The combined results from Datasets A and B demonstrate that the model exhibits excellent predictive capabilities in scenarios with substantial dynamic variations, such as ramp and stair climbing. CWT provides significant advantages in time-frequency feature extraction. CWT effectively captures local frequency domain information across different gait patterns and provides high-resolution time-frequency features, thereby enhancing the input quality for the deep learning model. Simultaneously, the Bi-LSTM network effectively models time-series dependencies. Bi-LSTM enhances the model’s ability to characterize complex dynamic features through bidirectional modeling, capturing long-term dependencies in time series, thereby improving prediction accuracy.

The model’s performance varied significantly across different activity types, exhibiting distinct adaptability in different scenarios within Datasets A and B. Within Dataset A, the model demonstrated optimal performance in ramp climbing and treadmill walking scenarios. Conversely, model performance decreased in stair descent and flat ground walking scenarios, resulting in reduced adaptability to scenes with more complex dynamic variations. Within Dataset B, during flat ground walking, the model accurately captured the motion dynamics of the hip joint torque, achieving a PCC of 0.985 $\pm$ 0.003, indicating a strong correlation. However, predictions of ankle joint torque exhibited increased error variance, likely due to the slight motion amplitude; the model presents inherent challenges in estimating low torques.

Within Dataset A, the model’s performance varied significantly across different activity types, particularly between the stair descent and treadmill walking scenarios. Specifically, in the stair descent scenario, the model’s prediction error was notably higher, and the fluctuation ranges for ${R^2}$ and RMSE were greater compared to the treadmill walking scenario. This performance discrepancy might be attributed to several factors. First, stair descent involves more pronounced nonlinear dynamic characteristics, such as rapid gait pattern changes and abrupt or decelerations. These characteristics increase the complexity of lower limb joint torque time-frequency characteristics, making it challenging for traditional convolutional feature extraction to capture them fully. Second, IMU data is subject to more significant noise interference during stair descent, including sensor motion artifacts and potential error accumulation. This further reduces data quality and the stability of the time-frequency characteristics. In contrast, the dynamic characteristics of treadmill walking are more straightforward and regular, contributing to better model performance in this scenario.

Future research could focus on optimizing feature extraction, data acquisition, and sensor fixation to further enhance the model’s predictive capabilities in scenarios with low-dynamic characteristics and complex dynamic scenarios (*e.g*., stair descent). Regarding feature extraction, incorporating multimodal data, such as surface EMG and GRF, could further enrich input features and enhance the model’s robustness to dynamic variations. Additionally, optimizing the parameter selection of CWT, such as scale factor and window size, could improve the ability to capture low-frequency signals and enhance the model’s adaptability in low-dynamic scenarios, such as flat ground walking. Secondly, expanding the data sample size for complex scenarios and introducing noise processing techniques, such as adaptive filters, could improve data quality, reduce errors, and enhance the model’s adaptability to complex dynamic scenarios. Finally, optimizing the sensor fixation method, such as improving strap design and incorporating a real-time calibration mechanism, could effectively reduce motion artifact interference and ensure consistency and accuracy in data acquisition.

### Baseline model results

To demonstrate the advantages of the proposed model, we benchmarked its performance against eight established baseline models—ANN, 1D CNN, Conv2D, GRU, LSTM, TCN, time transformer, and TCN-LSTM within the stair climbing context of Dataset A. The comparative results are detailed in Appendix 7.

As shown in Table 10, the proposed model’s overall performance in the stair-climbing scenario of Dataset A was significantly superior to all baseline models. For ankle joint torque, the proposed model achieved an RMSE of $0.148 \pm 0.012\;{\mathrm{N}} \cdot {\mathrm{m/kg}}$, representing a 22.3% reduction compared to the best-performing LSTM model ( $0.191 \pm 0.009\;{\mathrm{N}} \cdot {\mathrm{m/kg}}$). The ${R^2}$ value was $0.939 \pm 0.009$, outperforming the LSTM baseline ( $0.899 \pm 0.008$).

**Table 10:** Performance comparison with baseline models in stair ascent scenario.

Model	Hip *R*^2^	Knee *R*^2^	Ankle *R*^2^	Hip RMSE	Knee RMSE	Ankle RMSE
ANN	0.808 $\pm$ 0.012	0.782 $\pm$ 0.015	0.822 $\pm$ 0.018	0.251 $\pm$ 0.008	0.261 $\pm$ 0.009	0.253 $\pm$ 0.014
1D CNN	0.777 $\pm$ 0.014	0.748 $\pm$ 0.014	0.758 $\pm$ 0.019	0.270 $\pm$ 0.008	0.281 $\pm$ 0.008	0.295 $\pm$ 0.014
LSTM	0.909 $\pm$ 0.007	0.880 $\pm$ 0.009	0.899 $\pm$ 0.008	0.172 $\pm$ 0.007	0.193 $\pm$ 0.007	0.191 $\pm$ 0.009
Time transformer	0.913 $\pm$ 0.008	0.893 $\pm$ 0.011	0.908 $\pm$ 0.010	0.169 $\pm$ 0.008	0.182 $\pm$ 0.009	0.182 $\pm$ 0.011
TCN-LSTM	0.905 $\pm$ 0.011	0.883 $\pm$ 0.014	0.900 $\pm$ 0.020	0.176 $\pm$ 0.009	0.190 $\pm$ 0.011	0.189 $\pm$ 0.020
Proposed	0.950 $\pm$ 0.005	0.933 $\pm$ 0.007	0.939 $\pm$ 0.009	0.122 $\pm$ 0.010	0.150 $\pm$ 0.010	0.148 $\pm$ 0.012

Table 10 reveals that the proposed model demonstrates superior overall performance to all baseline models in the stair climbing scenario using Dataset A. For instance, considering ankle joint torque, the proposed model achieves an RMSE of 0.148 $\pm$ 0.012 Nm/kg, which is 18.7% and 21.7% lower than time transformer (0.182 $\pm$ 0.011 Nm/kg) and TCN-LSTM (0.189 $\pm$ 0.020 Nm/kg), respectively. Concurrently, the proposed method exhibits a significantly higher $R^2$ value (0.939 $\pm$ 0.009) compared to time transformer (0.908 $\pm$ 0.010) and TCN-LSTM (0.900 $\pm$ 0.020). This higher *R*^2^ value suggests a superior capability of the proposed method in complex time series modeling.

Among the evaluated baseline models in the stair climbing scenario, LSTM and 1D CNN demonstrated comparatively stronger performance. LSTM, capitalizing on its capacity for long-range dependency modeling, effectively captures the dynamic characteristics inherent in time series signals to a notable extent. As a result, its RMSE and ${R^2}$ metrics generally surpass those of simpler models like ANN and TCN across different joints (hip, knee, and ankle). However, LSTM’s inherent unidirectionality, which limits it to capturing only forward temporal dependencies, prevents it from fully leveraging backward information. This constraint impacts its performance when dealing with complex dynamic patterns, making it less effective than our proposed Bi-LSTM model.

In contrast to standard LSTM, Bi-LSTM, which is our proposed model, can concurrently capture both forward and backward temporal dependencies. This bidirectional capability proves particularly advantageous in modeling nonlinear gait patterns. In complex dynamic scenarios like stair climbing, Bi-LSTM’s bidirectional modeling significantly improves prediction accuracy. For instance, the knee joint ${R^2}$ for Bi-LSTM (Proposed) reached $0.933 \pm 0.007$, indicating its effectiveness in handling complex time-series dependencies and overcoming the unidirectionality limitations of standard LSTM. This represents a significant enhancement in prediction accuracy.

While the 1D CNN model also exhibited strong performance in extracting local features, its fixed receptive field limited its ability to model long-range temporal dependencies. Consequently, 1D CNN’s performance, as indicated by its knee joint ${R^2}$ of $0.880 \pm 0.009$, was notably lower than our proposed Bi-LSTM model’s knee joint ${R^2}$ of $0.933 \pm 0.007$. This highlights the superior capability of Bi-LSTM in capturing complex temporal dynamics for accurate motion prediction in challenging scenarios like stair ascent.

The performance of ANN and TCN was comparatively limited in the stair-climbing scenario. Without fully utilizing time-series information, ANN’s reliance on static feature extraction resulted in an RMSE of $0.251 \pm 0.008\;{\mathrm{N}} \cdot {\mathrm{m/kg}}$. Although TCN attempts to capture long-range dependencies by expanding the convolution window, its capacity to model temporal dynamics remains limited. Consequently, it was insufficient in processing complex actions such as stair climbing, with a PCC value of only $0.870 \pm 0.011$. Overall, while convolutional models (such as 1D CNN and TCN) effectively extract local features, their fixed receptive fields limit their capacity to capture long-range dependencies in time series. Additionally, ANN’s lack of dynamic feature extraction constrained its performance. Therefore, Bi-LSTM more effectively models the dynamic characteristics over long periods, whereas the fixed receptive fields of convolutional models limit their capacity to capture global temporal information.

Within the transformer family, the time transformer exhibited a slightly superior *R*^2^ index (0.913 $\pm$ 0.008) compared to LSTM (0.909 $\pm$ 0.007), suggesting a marginal advantage in capturing long-range dependencies. Nevertheless, its RMSE (0.169 $\pm$ 0.008 Nm/kg) remained considerably higher than our proposed model’s (0.122 $\pm$ 0.010 Nm/kg), indicating a notable prediction error. Moreover, the inherent O( ${n^2}$) computational complexity of the time transformer poses a significant limitation to its practical application, especially in the real-time processing of high-dimensional time series data. Conversely, our approach integrates CWT-based time-frequency feature extraction, 1D Conv ResNet for deep feature abstraction, and MHSA-Bi-LSTM for time series modeling. This synergistic combination reduces the computational complexity to O( $n\log n$) and enhances prediction accuracy, thereby achieving a superior balance between computational efficiency and prediction performance. Regarding the TCN-LSTM hybrid model, despite the enhanced time series modeling capabilities from the TCN-LSTM architecture, its *R*^2^ (0.900 $\pm$ 0.020) and RMSE (0.189 $\pm$ 0.020) metrics remained inferior to our proposed method, indicating suboptimal prediction accuracy and robustness in complex gait patterns. The inherent hierarchical structure of TCN-LSTM may contribute to attenuating long-term dependency information during propagation, potentially compromising prediction accuracy and rendering it less adaptable to the nonlinear dynamics of gait patterns compared to our proposed method.

### Statistical significance test

An independent samples $t$-test was employed to statistically analyze differences in model performance, evaluating the significance of the proposed model’s improvement. Key evaluation metrics—including RMSE, ${R^2}$, and MAE—were compared across different model architectures. The null hypothesis ( ${H_0}$) posited no significant difference between the proposed model and baseline models (LSTM & 1D CNN), while the alternative hypothesis ( ${H_1}$) asserted statistically significant improvements ( $\alpha = 0.05$). Statistical tests were conducted in Dataset A’s stair-climbing scenario, with evaluations of the proposed model, LSTM, and 1D CNN architectures over 20 iterations. The results are summarized in Table 11.

**Table 11:** Statistical significance test of model performance indicators in the stair climbing scenario.

Metric	Baseline model	Proposed (Avg)	Baseline (Avg)	t-value	*p*-value	Significance
RMSE	LSTM	0.176 $\pm$ 0.010	0.151 $\pm$ 0.011	3.91	0.0012	Significance
	1D CNN	0.287 $\pm$ 0.013	0.151 $\pm$ 0.011	7.86	0.00003	Significance
*R* ^2^	LSTM	0.914 $\pm$ 0.007	0.930 $\pm$ 0.006	3.42	0.0028	Significance
	1D CNN	0.766 $\pm$ 0.009	0.930 $\pm$ 0.006	9.12	0.00001	Significance
MAE	LSTM	0.124 $\pm$ 0.005	0.112 $\pm$ 0.004	3.71	0.0016	Significance
	1D CNN	0.211 $\pm$ 0.007	0.112 $\pm$ 0.004	8.34	0.00002	Significance

The results indicated that the proposed model outperformed the LSTM and 1D CNN architectures across all key evaluation metrics. The RMSE value for the proposed model was significantly lower than those of LSTM (t = 3.91, *p* = 0.0012) and 1D CNN (t = 7.86, *p* = 0.00003), confirming the proposed model’s significant reduction in prediction error. Similarly, the ${R^2}$ value for the proposed model was significantly higher than those of LSTM (t = 3.42, *p* = 0.0028) and 1D CNN (t = 9.12, *p* = 0.00001), indicating the model’s improved accuracy and reliability. Furthermore, the MAE value for the proposed model was also significantly lower than those of LSTM (t = 3.71, *p* = 0.0016) and 1D CNN (t = 8.34, *p* = 0.00002), further demonstrating the model’s robustness in minimizing absolute error. To verify the applicability of the t-test, the Shapiro-Wilk normality test was performed on the distributions of RMSE and ${R^2}$. The results confirmed that all datasets exhibited a normal distribution (*p* > 0.05), thus satisfying the prerequisites for applying the t-test.

### Ablation experiment

An ablation study was conducted to verify the contribution of each module to the proposed model’s overall performance. The ablation study involved the progressive removal or replacement of key modules, including CWT, 1D Conv ResNet, and Bi-LSTM, to evaluate each module’s specific impact on model performance. The study was conducted using a ramp-up scenario, and performance changes were evaluated using four metrics: RMSE, R^2^, MAE, and PCC. The results of the ablation study are presented in Table 12.

**Table 12:** Performance indicators under various configurations.

Moment	Indicators	1D Conv ResNet	MHSA	Only LSTM	Bi-LSTM	CWT
Hip flexion	${R^2}$	0.861 $\pm$ 0.054	0.897 $\pm$ 0.027	0.857 $\pm$ 0.043	0.871 $\pm$ 0.034	0.858 $\pm$ 0.049
	RMSE	0.216 $\pm$ 0.049	0.188 $\pm$ 0.025	0.222 $\pm$ 0.032	0.211 $\pm$ 0.028	0.220 $\pm$ 0.039
	MAE	0.169 $\pm$ 0.039	0.139 $\pm$ 0.018	0.165 $\pm$ 0.023	0.172 $\pm$ 0.024	0.165 $\pm$ 0.029
Knee angle	${R^2}$	0.778 $\pm$ 0.049	0.852 $\pm$ 0.038	0.783 $\pm$ 0.050	0.811 $\pm$ 0.034	0.790 $\pm$ 0.050
	RMSE	0.255 $\pm$ 0.029	0.208 $\pm$ 0.027	0.252 $\pm$ 0.029	0.236 $\pm$ 0.022	0.248 $\pm$ 0.031
	MAE	0.194 $\pm$ 0.021	0.155 $\pm$ 0.017	0.186 $\pm$ 0.019	0.187 $\pm$ 0.019	0.193 $\pm$ 0.028
	PCC	0.886 $\pm$ 0.028	0.927 $\pm$ 0.018	0.891 $\pm$ 0.027	0.923 $\pm$ 0.018	0.898 $\pm$ 0.024
Ankle angle	${R^2}$	0.892 $\pm$ 0.038	0.901 $\pm$ 0.022	0.865 $\pm$ 0.027	0.855 $\pm$ 0.038	0.865 $\pm$ 0.035
	RMSE	0.204 $\pm$ 0.042	0.197 $\pm$ 0.023	0.230 $\pm$ 0.026	0.238 $\pm$ 0.032	0.229 $\pm$ 0.032
	MAE	0.136 $\pm$ 0.029	0.130 $\pm$ 0.012	0.144 $\pm$ 0.014	0.186 $\pm$ 0.033	0.155 $\pm$ 0.025
	PCC	0.946 $\pm$ 0.019	0.952 $\pm$ 0.011	0.933 $\pm$ 0.013	0.939 $\pm$ 0.015	0.935 $\pm$ 0.016

The ablation study results indicated that the complete model achieved optimal performance in the ramp-ascending scenario, with an RMSE of 0.148 $\pm$ 0.008, an *R*^2^ of 0.933 $\pm$ 0.007, and a PCC of 0.966 $\pm$ 0.003, demonstrating the effectiveness of the proposed architecture. Removing the CWT led to a significant decrease in model performance, with the RMSE increasing to 0.193 $\pm$ 0.012 and the R^2^ decreasing to 0.876 $\pm$ 0.015. These results indicate that CWT is key in extracting multi-scale time-frequency features from gait signals. Compared to using only the original IMU signal, CWT provides the model with richer and more discriminative input data by capturing features across different frequency ranges, thus significantly improving prediction accuracy.

Removing the 1D Conv ResNet increased RMSE to 0.176 $\pm$ 0.011 and caused a decrease in R^2^ to 0.910 $\pm$ 0.009. The 1D Conv ResNet effectively enhances local time-frequency domain feature modeling capabilities during feature extraction and captures the change patterns of IMU signals across different time-frequency scales. The convolutional structure not only extracts short-term dependencies but also improves the capacity to capture complex dynamic information through hierarchical feature extraction and stabilizes gradient flow through residual connections, thus enhancing feature representation and improving model optimization efficiency.

Removing the Bi-LSTM resulted in a relatively small performance degradation, yet it still affected overall prediction capability. The RMSE increased to 0.162 $\pm$ 0.010, and the R^2^ decreased to 0.919 $\pm$ 0.010. Compared to unidirectional LSTM, Bi-LSTM can simultaneously model both forward and backward time-series information, effectively integrating the information from the complete time series. Thus, it improves model prediction accuracy and demonstrates higher robustness in dynamic and complex scenarios.

Ablation study results, detailed in Appendix 8, demonstrate a significant performance enhancement with the progressive integration of 1D Conv ResNet, MHSA, and Bi-LSTM modules, thereby validating the efficacy of our feature fusion strategy. Specifically, 1D Conv ResNet excels at extracting deep features, MHSA effectively reinforces global dependencies, and Bi-LSTM optimizes time series modeling. These three components synergistically enhance the accuracy of lower limb torque estimation. The baseline model, comprising CWT and 1D Conv ResNet, is limited to capturing only local features and lacks inherent time series modeling capabilities. This limitation manifests as a low *R*^2^ (knee joint: 0.743 $\pm$ 0.052) and a high RMSE. Integrating MHSA enhances global information interaction and improves PCC. However, the marginal reduction in RMSE suggests that relying solely on the attention mechanism is insufficient for accurately predicting the intricate dynamics of lower limb motion. The complete model, incorporating CWT, 1D Conv ResNet, MHSA, and Bi-LSTM, further refines temporal dependency modeling *via* Bi-LSTM. This refinement leads to a substantial increase in R^2^ to 0.942 $\pm$ 0.028 (ankle), 0.885 $\pm$ 0.071 (knee), and 0.948 $\pm$ 0.034 (hip), coupled with a significant RMSE reduction, demonstrating optimal prediction accuracy and robustness. The observed performance gains from feature fusion stem from the complementary interplay of these distinct modules rather than any singular enhancement. The complete model achieves a notable performance leap in complex dynamic scenarios by effectively integrating the time-frequency features extracted by CWT with the sophisticated feature modeling capabilities of the deep learning modules.

## Discussion

This study introduces a novel hybrid framework integrating CWT with deep learning architectures to estimate lower limb joint torque accurately. Compared to traditional time or frequency domain approaches, CWT enables multi-scale local and global feature representations in both time and frequency domains, effectively capturing transient vibrations and dynamic trends in IMU data while providing more informative input features for deep learning networks. This approach is computationally efficient for highly dynamic, nonlinear motion signals, such as those encountered during ramp walking and stair climbing. Furthermore, CWT’s multi-scale analysis capability facilitates the effective separation of high-frequency noise and enhancement of low-frequency dynamic changes, thereby providing a more robust feature basis for lower limb torque estimation.

Compared to existing deep learning methods (TCNs and CNNs), TCNs capture temporal dependencies. However, they are limited by their fixed receptive fields when modeling irregular motion patterns. At the same time, CNNs excel at spatial feature extraction but fail to capture long-term temporal dependencies inherent in gait data. By integrating CWT, MHSA, and Bi-LSTM, our model addresses the limitations of traditional methods in feature extraction, temporal modeling, and information focusing. Specifically, CWT is a preprocessing step that transforms raw IMU data into time-frequency domain features, preserving critical time and frequency information while enhancing the model’s capability to process non-stationary signals. The MHSA mechanism refines the model’s attention to time-frequency features, allowing identification and emphasis of key nodes in motion sequences, thereby enhancing estimation accuracy and robustness for complex gait data. The Bi-LSTM network models long-term temporal dependencies through bidirectional information flow, enabling consideration of both historical and future states, thus improving adaptability to temporal dynamics. When processing complex lower limb motion patterns, our model demonstrates superior generalization and stability compared to traditional methods.

Experimental results demonstrate significant performance improvements achieved by the proposed model. For ramp ascent and treadmill walking scenarios in Dataset A, the model achieved *R*^2^ values of 0.957 and 0.959, respectively, significantly outperforming the baseline LSTM model (*R*^2^ = 0.909). Evaluation of Dataset B further validates the model’s effectiveness. Additionally, experimental results indicate an end-to-end inference time of 152.6 milliseconds for real-time analysis, satisfying real-time processing requirements exceeding 100 Hz. Compared to methods such as LSTM ( $O(t \cdot {h^2})$), our approach achieves a computational complexity of $O(n \cdot w \cdot d)$, representing a 37.2% improvement in computational efficiency.

Comparative analysis reveals that our method achieves comparable or superior estimation accuracy using only a single IMU data input compared to existing literature. For instance, using multimodal data, Altai et al.’s (2023) XCM deep network achieved an RMSE of 0.046 $\pm$ 0.013 N $\cdot$m/kg. In comparison, Camargo et al. (2021) reported a MAE of 0.06 $\pm$ 0.02 N $\cdot$m/kg by fusing IMU and ground reaction force data. However, these methods require multi-sensor data fusion, increasing equipment complexity and cost while limiting practical deployment. In contrast, our model achieves comparable accuracy using only a single IMU sensor, significantly reducing hardware requirements and data acquisition complexity while enhancing feasibility for portable applications.

This study significantly enhances lower limb joint torque estimation accuracy and model robustness by integrating CWT, Bi-LSTM, and MHSA. Compared to existing methods, the proposed model demonstrates significant advantages in lightweight design, reduced data dependency, and practical application potential, offering novel solutions for portable rehabilitation devices, human-computer interaction systems, and sports biomechanics analysis. Future research will address challenges, including data noise, sensor drift, and individualized modeling in complex scenarios, further advancing the technology’s practical applications in clinical, industrial, and sports health monitoring domains.

### Limitations of the study and future work

While the proposed hybrid model combining CWT and deep learning demonstrates improved lower limb joint torque estimation, several limitations require further investigation. First, data collection and model validation were conducted under controlled laboratory conditions using standardized gait patterns from healthy participants. While controlled environments facilitate preliminary validation, real-world scenarios (*e.g*., outdoor walking, mountain biking, running) introduce challenges, including complex terrain, non-standard movements (sharp turns, rapid accelerations), and environmental interference that may adversely affect data quality. Furthermore, IMU sensor measurements in uncontrolled environments are susceptible to noise, signal drift, and inter-subject variability, potentially limiting model generalizability. Future work should expand datasets to include diverse real-world scenarios and enhance model robustness through training-phase interventions, including data augmentation (simulated sensor drift, random noise, dynamic artifacts) and domain adaptation methods for improved noise tolerance. Field deployment with *in situ* data collection is required to validate model robustness in uncontrolled settings.

To rigorously assess model robustness, this study systematically simulated potential real-world data anomalies through a data augmentation strategy. Specifically, three common error scenarios were simulated: Gaussian noise to represent environmental interference, sensor offset to mimic zero drift, and sensor misalignment to emulate wearable sensor displacement. Specifically, Gaussian noise with zero mean and a standard deviation of 0.05 was injected into IMU data. A fixed offset of 0.1 was introduced, and sensor misalignment was simulated by applying a 5° rotation *via* a 3D rotation matrix. These perturbations effectively replicated typical noise patterns encountered in real-world settings. During the evaluation phase, model performance was assessed across original, Gaussian noise-augmented, offset-perturbed, and misalignment-perturbed data.

As detailed in Appendix 9, experimental findings reveal that the model sustains high prediction accuracy across diverse perturbed conditions. Even when subjected to noise and offset, *R*^2^ consistently exceeds 0.96, and PCC surpasses 0.98, unequivocally demonstrating robust resilience to sensor-induced errors. While this study has rigorously evaluated the model’s generalization capability through simulation-based noise injection, validation in authentic wearable settings remains an essential next step. Future research will prioritize real-world data acquisition and experimentation, employing wearable IMU devices to capture *in situ* gait data. Further refinement of error compensation algorithms will be undertaken to bolster model reliability, stability, and generalizability within practical, uncontrolled environments. Field deployment of the system for data collection in ecologically valid scenarios will be crucial for definitive robustness validation under real-world complexities.

Second, IMU sensor errors and drift constitute critical constraints on model performance. Sensor placement inaccuracies (position/orientation offsets) introduce measurement errors, while long-term monitoring drift leads to the accumulation of low-frequency noise that compromises time-frequency feature extraction and modeling precision. To address these limitations, implementation of high-precision calibration algorithms (Kalman filtering, complementary filtering) could mitigate measurement errors. Developing standardized sensor placement protocols (specifying position, orientation, and attachment tension) would reduce installation-induced data variability. Incorporating training-phase perturbation strategies (simulated positional shifts, random noise injection) could enhance model robustness against sensor errors.

Third, inter-individual gait variability presents challenges to model generalizability. While trained and validated on healthy individuals’ standardized gait data, the model may not adequately capture natural gait variations arising from age, sex, health conditions, and movement habits in real-world applications. Furthermore, pathological gait patterns (post-stroke or Parkinsonian gait) exhibit strong nonlinearity and irregularity, yet model performance under these conditions remains insufficiently validated. Future work should integrate transfer learning or domain adaptation techniques to address these limitations, enabling model fine-tuning with limited subject-specific data to enhance gait pattern adaptability. Concurrently, expanding datasets to encompass diverse age groups, biological sexes, and pathological populations would improve model generalizability across clinical and real-world scenarios (Sánchez et al., 2017).

Fourthly, the dataset employed in this study exhibits an intrinsic class imbalance characterized by pronounced disparities in sample distributions across various gait patterns. For instance, the abundance of gait cycles in flat ground walking mode substantially outweighs that in ramp climbing mode, potentially inducing class bias during the training phase. This categorical imbalance could compromise the model’s training efficacy and generalization capacity. In the current iteration of this study, we opted not to employ sampling strategies or class weighting techniques to mitigate the aforementioned class imbalance. Our initial research focused on validating the efficacy of the proposed CWT and deep learning hybrid framework, alongside evaluating its overall performance in lower limb joint torque estimation. Nonetheless, we fully acknowledge the potential ramifications of class imbalance and are committed to systematically addressing this issue in future research endeavors. Future research directions will encompass optimizations at both the data and algorithmic levels. At the data level, we will leverage data augmentation strategies, including time warping, additive noise injection, and interpolation-based augmentation, to augment sample sizes for minority classes. This will promote a more balanced data distribution and enhance the model’s generalization across all gait patterns. Concurrently, we will introduce a class-weighted loss function at the algorithmic level and employ random oversampling to refine the model’s generalization capabilities across all gait modes. These data- and algorithm-level refinements are anticipated to enhance the model’s robustness and generalization further, enabling superior performance in increasingly complex and diverse real-world application scenarios.

Furthermore, the computational demand of the model represents a critical constraint that warrants careful consideration. While the proposed hybrid model demonstrates notable performance advantages, computational complexity analysis, and experimental evaluations indicate that it achieves a favorable trade-off between accuracy and real-time capability. Experimentally, on an NVIDIA RTX 3090 GPU, the end-to-end inference time per gait cycle is measured at 152.6 milliseconds. While preliminarily satisfying the real-time constraint for a 100 Hz sampling rate, this latency necessitates further optimization for wearable applications. Given the inherent computational limitations of wearable devices and our framework’s reliance on CWT and MHSA modules, the inference overhead may present challenges for on-device deployment. Future research will prioritize the exploration of model light-weighting techniques. Specific strategies will encompass model pruning and quantization to reduce parameter counts and computational precision demands; knowledge distillation to transfer knowledge from complex models to lightweight architectures; development of efficient CWT approximation algorithms, such as the fast wavelet transform; and investigation of hardware acceleration solutions, including FPGA and application-specific integrated circuit (ASIC) implementations, to leverage parallel processing for accelerated inference. We anticipate successfully deploying our framework on resource-constrained edge devices by implementing these light weighting strategies and hardware optimizations. This will pave the way for realizing truly wearable, real-time lower limb torque monitoring in ambulatory settings.

The developed model demonstrates substantial industrial applicability, particularly for exoskeleton systems and human-robot collaboration technologies. Accurate joint torque estimation is critical for enhancing robotic responsiveness and safety in human-robot collaboration and exoskeleton applications. The framework enables dynamic torque feedback for exoskeletons and collaborative robots in industrial manufacturing, eldercare, and rehabilitation settings, enhancing human-robot interaction adaptability and operational efficiency. For instance, elderly lower-limb assistive devices that utilize accurate torque estimation can enhance balance control during ambulation and stair negotiation, subsequently improving mobility and quality of life (Liang et al., 2024; Abdullahi, Haruna & Chaichaowarat, 2024; Huo et al., 2014; Shi et al., 2019). Compared to multi-sensor fusion approaches, our method enhances practicality and cost-effectiveness through reduced sensor dependency.

In sports biomechanics, this technology offers potential for athlete performance optimization through real-time monitoring of joint kinetics and movement kinematics, providing data-driven feedback to mitigate injury risks and enhance training efficacy. Unlike traditional motion analysis, which requires force platforms or optical systems, IMU-based solutions provide portable and cost-effective alternatives enabling on-site analysis in athletic environments.

For rehabilitation medicine and intelligent health monitoring, the model’s real-time feedback precision makes it particularly suitable for wearable implementations. Real-time biomechanical monitoring enables dynamic adjustment of rehabilitation protocols to enhance gait stability and motor function recovery post-surgery or during convalescence. The model’s minimal data and hardware requirements lower implementation barriers, extending its applicability to resource-limited rehabilitation settings. When integrated with low-cost IMUs and cloud computing, the system enables telerehabilitation and personalized health management through real-time fall risk assessment and health status monitoring, particularly benefitting geriatric populations.

Future research directions include (1) enhancing model robustness through dataset expansion and architectural optimization, (2) implementing adaptive calibration strategies, and (3) advancing applications in industrial, rehabilitation, sports, and health monitoring domains to establish reliable lower-limb torque estimation frameworks.

## Conclusions

Addressing the high costs associated with optical motion capture systems and force platforms in gait analysis, this study introduces a hybrid model predicated on continuous wavelet transform (CWT) and deep learning. This model leverages single inertial measurement unit (IMU) data to achieve accurate estimation of hip, knee, and ankle joint torques. Experimental results demonstrate superior performance over conventional methods across key metrics (*R*^2^ = 0.96, RMSE = 0.16 N $\cdot$m/kg, MAE = 0.15 N $\cdot$m/kg), thereby demonstrating its high accuracy and robustness. However, potential discrepancies between IMU data acquired in controlled laboratory environments and naturalistic walking conditions may constrain the model’s generalizability, necessitating further validation. Future research endeavors should prioritize the assessment of the model’s adaptability within ecologically valid scenarios, encompassing data from real-world ambulation contexts such as level-ground walking, stair negotiation, and ramp traversal. The core innovation of this research lies in the synergistic integration of CWT with deep learning architectures to realize high-precision lower limb joint torque estimation utilizing a single IMU sensor configuration, thereby substantially reducing hardware complexity compared to multimodal systems. In contrast to multimodal data fusion approaches, this framework maintains comparable accuracy while offering enhanced practicality and cost-effectiveness, presenting a novel solution for portable rehabilitation devices, human-computer interaction technologies, and sports biomechanics analysis. Future research directions encompass expanding the datasets to incorporate a broader spectrum of real-world scenarios and pathological gait data, refining sensor error mitigation techniques, and exploring the model’s deployability on low-power edge computing platforms.

## Supplemental Information

10.7717/peerj-cs.2888/supp-1 Supplemental Information 1 Appendices.

10.7717/peerj-cs.2888/supp-2 Supplemental Information 2 The core procedures of this study.

## Additional Information and Declarations

### Data Availability

The following information was supplied regarding data availability:

The lower limb biomechanics datasets are available at Mendeley Data:

- Camargo, Jonathan (2021), “Data repository for Camargo, et. al. A comprehensive, open-source dataset of lower limb biomechanics. Part 1 of 3”, Mendeley Data, V1, doi: 10.17632/fcgm3chfff.1.

- Camargo, Jonathan (2021), “Data repository for Camargo, et. al. A comprehensive, open-source dataset of lower limb biomechanics. Part 2 of 3”, Mendeley Data, V1, doi: 10.17632/k9kvm5tn3f.1.

- Camargo, Jonathan (2021), “Data repository for Camargo, et. al. A comprehensive, open-source dataset of lower limb biomechanics. Part 3 of 3”, Mendeley Data, V1, doi: 10.17632/jj3r5f9pnf.1.

A Human Lower-Limb Biomechanics and Wearable Sensors Dataset During Cyclic and Non-Cyclic Activities is available at Georgia Tech Digital Repository: https://doi.org/10.35090/gatech/70296.

The full source code and copy scripts are available at GitHub and Zenodo, which also include detailed instructions for reproducing the results:

- https://github.com/Hihubxu/Estimation-of-Lower-Limb-Torque.

- https://doi.org/10.5281/zenodo.14826881.

The relevant programs, the processed data files, README files, and detailed instructions for utilizing third-party data to ensure comprehensive understanding and replication of the research findings are available in the Supplemental File.
