# Supplementary material for: Estimation of lower limb torque: a novel hybrid method based on continuous wavelet transform and deep learning approach
Source: PeerJ Comput Sci. 2025 May 30;11:e2888. doi: 10.7717/peerj-cs.2888 (PMC12192784; doi:10.7717/peerj-cs.2888)
Supplement: Supplemental Information 1 [file peerj-cs-11-2888-s001.docx]

Appendix 1：Comparative Experimental Results of CWT, AF and EMD

| method | R² | MAE | PCC | SNR (dB) | MSE |
| --- | --- | --- | --- | --- | --- |
| CWT | 0.922 | 0.113 ± 0.007 | 0.961 ± 0.005 | -3.057 | 0.920 |
| AF | 0.951 | 0.112 ± 0.010 | 0.976 ± 0.007 | -2.859 | 0.682 |
| EMD | 0.859 | 0.146 ± 0.021 | 0.929 ± 0.029 | -13.588 | 8.050 |

Appendix 2：Comparison of model performance with different mother wavelets

| Mother wavelet type | RMSE | R² | PCC |
| --- | --- | --- | --- |
| Morlet | 0.191 ± 0.031 | 0.874 ± 0.041 | 0.939 ± 0.020 |
| Mexican Hat | 0.219 ± 0.044 | 0.832 ± 0.070 | 0.915 ± 0.039 |
| Daubechies | 0.261 ± 0.040 | 0.766 ± 0.068 | 0.878 ± 0.037 |

Appendix 3：The impact of different ω₀ values on model performance

| ω₀ | RMSE | R² | PCC |
| --- | --- | --- | --- |
| 4 | 0.206 ± 0.042 | 0.852 ± 0.058 | 0.927 ± 0.028 |
| 5 | 0.188 ± 0.064 | 0.868 ± 0.095 | 0.935 ± 0.050 |
| 6 | 0.220 ± 0.057 | 0.826 ± 0.088 | 0.912 ± 0.047 |
| 7 | 0.181 ± 0.031 | 0.874 ± 0.041 | 0.939 ± 0.020 |
| 8 | 0.223 ± 0.058 | 0.822 ± 0.094 | 0.911 ± 0.048 |

Appendix 4： Performance comparison of regularization technique combinations

| Technical configuration | RMSE(Nm/kg) | R² | PCC |
| --- | --- | --- | --- |
| No regularization | 0.214 ± 0.057 | 0.835 ± 0.080 | 0.916 ± 0.042 |
| Dropout only | 0.201 ± 0.034 | 0.861 ± 0.046 | 0.932 ± 0.023 |
| BN only | 0.196 ± 0.032 | 0.868 ± 0.042 | 0.935 ± 0.020 |
| Dropout + BN + L2 | 0.193 ± 0.064 | 0.862 ± 0.093 | 0.931 ± 0.048 |

Appendix 5：Details of the training and test datasets

| Dataset name | Activity Mode | Dataset | Training set (70\%) | Validation set (10\%) | Test set (20\%) |
| --- | --- | --- | --- | --- | --- |
| Dataset A | Flat ground | 732 | 511 | 73 | 148 |
|  | Ramp ascent | 309 | 216 | 31 | 62 |
|  | Ramp descent | 467 | 327 | 46 | 94 |
|  | Stair ascent | 797 | 558 | 79 | 160 |
|  | Stair descent | 826 | 578 | 82 | 166 |
|  | Treadmill | 15292 | 10704 | 1529 | 3059 |
| Dataset B | Flat ground | 990 | 693 | 99 | 198 |
|  | Ramp | 388 | 271 | 39 | 78 |

Appendix 6：Performance indicators of the model in six scenarios

| Moment | Indicators | Ramp ascent | Ramp descent | Stair ascent | Stair descent | Treadmill | Flat ground |
| --- | --- | --- | --- | --- | --- | --- | --- |
| Hip flexion moment | R² | 0.957 ± 0.007 | 0.907 ± 0.022 | 0.933 ± 0.007 | 0.811 ± 0.024 | 0.959 ± 0.005 | 0.898 ± 0.015 |
|  | RMSE | 0.122 ± 0.010 | 0.164 ± 0.020 | 0.148 ± 0.008 | 0.236 ± 0.015 | 0.121 ± 0.006 | 0.178 ± 0.013 |
|  | MAE | 0.091 ± 0.006 | 0.119 ± 0.011 | 0.110 ± 0.005 | 0.183 ± 0.012 | 0.086 ± 0.003 | 0.132 ± 0.008 |
|  | PCC | 0.979 ± 0.003 | 0.953 ± 0.011 | 0.966 ± 0.003 | 0.903 ± 0.013 | 0.979 ± 0.002 | 0.949 ± 0.008 |
| Knee angle moment | R² | 0.903 ± 0.047 | 0.936 ± 0.024 | 0.927 ± 0.010 | 0.936 ± 0.007 | 0.940 ± 0.006 | 0.81 ± 0.069 |
|  | RMSE | 0.166 ± 0.015 | 0.154 ± 0.025 | 0.150 ± 0.010 | 0.158 ± 0.009 | 0.131 ± 0.007 | 0.231 ± 0.025 |
|  | MAE | 0.119 ± 0.018 | 0.106 ± 0.007 | 0.109 ± 0.006 | 0.112 ± 0.006 | 0.086 ± 0.004 | 0.173 ± 0.015 |
|  | PCC | 0.952 ± 0.024 | 0.968 ± 0.012 | 0.963 ± 0.005 | 0.968 ± 0.004 | 0.970 ± 0.003 | 0.889 ± 0.018 |
| Ankle angle moment | R² | 0.955 ± 0.021 | 0.934 ± 0.021 | 0.939 ± 0.009 | 0.914 ± 0.009 | 0.978 ± 0.004 | 0.891 ± 0.054 |
|  | RMSE | 0.130 ± 0.019 | 0.158 ± 0.025 | 0.148 ± 0.012 | 0.197 ± 0.010 | 0.094 ± 0.009 | 0.203 ± 0.050 |
|  | MAE | 0.081 ± 0.012 | 0.101 ± 0.007 | 0.096 ± 0.007 | 0.129 ± 0.007 | 0.054 ± 0.003 | 0.132 ± 0.010 |
|  | PCC | 0.978 ± 0.011 | 0.967 ± 0.011 | 0.969 ± 0.005 | 0.957 ± 0.005 | 0.989 ± 0.002 | 0.945 ± 0.015 |

Appendix 7：Performance indicators of each deep learning model in the stair climbing scenario

| Moment | Indicators | ANN | Conv 1D | Conv 2D | GRU | LSTM | TCN | Time Transformer | TCN-LSTM |
| --- | --- | --- | --- | --- | --- | --- | --- | --- | --- |
| Hip flexion moment | R² | 0.808 ± 0.012 | 0.777 ± 0.014 | 0.812 ± 0.013 | 0.777 ± 0.015 | 0.909 ± 0.007 | 0.756 ± 0.019 | 0.913 ± 0.008 | 0.905 ± 0.011 |
|  | RMSE | 0.251± 0.008 | 0.270± 0.008 | 0.248 ± 0.009 | 0.270 ± 0.009 | 0.172 ± 0.007 | 0.282 ± 0.011 | 0.169 ± 0.008 | 0.176 ± 0.009 |
|  | MAE | 0.191 ± 0.006 | 0.206 ± 0.007 | 0.188 ± 0.007 | 0.206 ± 0.007 | 0.129 ± 0.005 | 0.219 ± 0.008 | 0.126 ± 0.006 | 0.129 ± 0.007 |
|  | PCC | 0.899 ± 0.007 | 0.882 ± 0.008 | 0.902 ± 0.007 | 0.882 ± 0.008 | 0.954 ± 0.004 | 0.870 ± 0.011 | 0.956 ± 0.004 | 0.952 ± 0.006 |
| Knee angle moment | R² | 0.782 ± 0.015 | 0.748 ± 0.014 | 0.783 ± 0.015 | 0.737 ± 0.016 | 0.880 ± 0.009 | 0.724 ± 0.020 | 0.893 ± 0.011 | 0.883 ± 0.014 |
|  | RMSE | 0.261 ± 0.009 | 0.281 ± 0.008 | 0.260 ± 0.009 | 0.287 ± 0.009 | 0.193 ± 0.007 | 0.294 ± 0.011 | 0.182 ± 0.009 | 0.190 ± 0.011 |
|  | MAE | 0.202 ± 0.007 | 0.217 ± 0.007 | 0.201 ± 0.007 | 0.223 ± 0.008 | 0.142 ± 0.005 | 0.230 ± 0.008 | 0.131 ± 0.005 | 0.137 ± 0.009 |
|  | PCC | 0.884 ± 0.008 | 0.865 ± 0.008 | 0.885 ± 0.008 | 0.859 ± 0.009 | 0.939 ± 0.005 | 0.851 ± 0.012 | 0.946 ± 0.006 | 0.940 ± 0.007 |
| Ankle angle moment | R² | 0.822 ± 0.018 | 0.758 ± 0.019 | 0.815 ± 0.014 | 0.742 ± 0.018 | 0.899 ± 0.008 | 0.742 ± 0.013 | 0.908 ± 0.010 | 0.900 ± 0.020 |
|  | RMSE | 0.253 ± 0.014 | 0.295 ± 0.014 | 0.258 ± 0.011 | 0.305 ± 0.012 | 0.191 ± 0.009 | 0.305 ± 0.009 | 0.182 ± 0.011 | 0.189 ± 0.020 |
|  | MAE | 0.188 ± 0.009 | 0.216 ± 0.009 | 0.185 ± 0.007 | 0.215 ± 0.007 | 0.128 ± 0.006 | 0.229 ± 0.005 | 0.119 ± 0.006 | 0.122 ± 0.011 |
|  | PCC | 0.907 ± 0.010 | 0.871 ± 0.011 | 0.903 ± 0.008 | 0.862 ± 0.010 | 0.948 ± 0.004 | 0.862 ± 0.008 | 0.953 ± 0.005 | 0.949 ± 0.010 |

Appendix 8：Performance indicators under various configurations

| Model | Moment | R² | RMSE (Nm/kg) | MAE | PCC |
| --- | --- | --- | --- | --- | --- |
| CWT + 1D Conv ResNet | Ankle angle moment | 0.831 ± 0.026 | 0.259 ± 0.024 | 0.189 ± 0.011 | 0.916 ± 0.014 |
|  | Knee angle moment | 0.743 ± 0.052 | 0.275 ± 0.029 | 0.215 ± 0.017 | 0.866 ± 0.031 |
|  | Hip flexion moment | 0.812 ± 0.052 | 0.255 ± 0.033 | 0.197 ± 0.021 | 0.906 ± 0.027 |
| CWT + 1D Conv ResNet + MHSA | Ankle angle moment | 0.840 ± 0.040 | 0.250 ± 0.032 | 0.221 ± 0.030 | 0.968 ± 0.008 |
|  | Knee angle moment | 0.769 ± 0.035 | 0.262 ± 0.020 | 0.219 ± 0.013 | 0.947 ± 0.023 |
|  | Hip flexion moment | 0.841 ± 0.031 | 0.235 ± 0.025 | 0.198 ± 0.022 | 0.974 ± 0.004 |
| CWT + 1D Conv ResNet + MHSA + Bi-LSTM | Ankle angle moment | 0.942 ± 0.028 | 0.148 ± 0.034 | 0.095 ± 0.023 | 0.972 ± 0.014 |
|  | Knee angle moment | 0.885 ± 0.071 | 0.178 ± 0.048 | 0.133 ± 0.039 | 0.943 ± 0.035 |
|  | Hip flexion moment | 0.948 ± 0.034 | 0.130 ± 0.036 | 0.100 ± 0.030 | 0.975 ± 0.017 |

Appendix 9：Evaluate the performance of the model in different environments

| Environment | R² | RMSE | MAE | PCC |
| --- | --- | --- | --- | --- |
| Original | 0.955 ± 0.019 | 0.116 ± 0.023 | 0.081 ± 0.017 | 0.978 ± 0.010 |
| Noisy | 0.965 ± 0.015 | 0.105 ± 0.018 | 0.074 ± 0.013 | 0.983 ± 0.008 |
| Sensor Bias | 0.971 ± 0.014 | 0.095 ± 0.017 | 0.064 ± 0.012 | 0.986 ± 0.007 |
| Misaligned | 0.968 ± 0.016 | 0.100 ± 0.019 | 0.070 ± 0.014 | 0.984 ± 0.008 |

(1) Original (original data source), Data without any noise or error treatment; (2) Noisy (Gaussian noise data), Adding Gaussian noise to IMU signals to simulate environment interference；(3) Sensor Bias (sensor offset), Artificially added fixed offset to simulate zero drift error；(4) Misaligned (sensor misalignment), Small angle rotation of IMU data to simulate wearing errors or inaccurate device installation。

Appendix 10. Continuous wavelet transform (CWT) representations of six thigh-mounted IMU axes for sensor-level discriminative analysis.


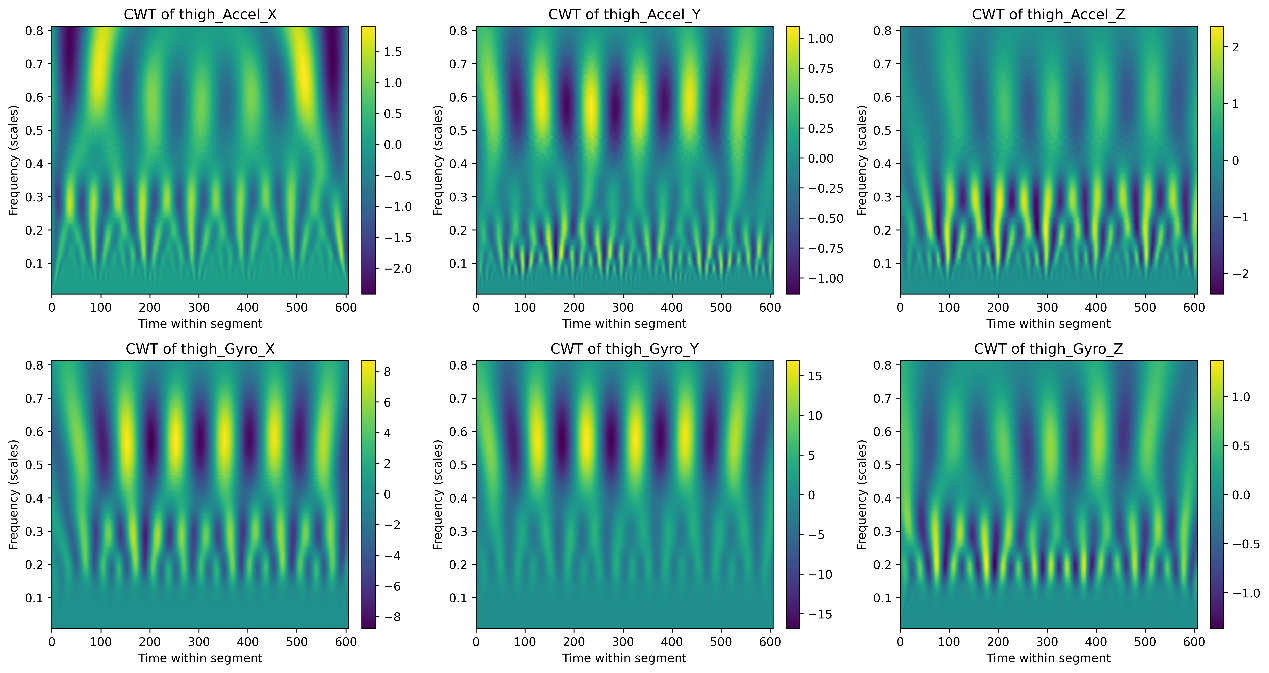


The CWT plots visualize the time–frequency patterns of six IMU channels mounted on the thigh. Among them, thigh_Accel_Y and thigh_Gyro_Y exhibit more distinct and concentrated spectral structures, suggesting stronger discriminative power for activity classification.

.
